# Electrochemical Nanoreactor Provides a Comprehensive View of Isocitrate Dehydrogenase Cancer‐drug Kinetics

**DOI:** 10.1002/ange.202309149

**Published:** 2023-09-12

**Authors:** Ryan A. Herold, Christopher J. Schofield, Fraser A. Armstrong

**Affiliations:** ^1^ Inorganic Chemistry Laboratory Department of Chemistry University of Oxford South Parks Road Oxford OX1 3QR UK; ^2^ Department of Chemistry and the Ineos Oxford Institute for Antimicrobial Research University of Oxford Mansfield Road Oxford OX1 3QY UK

**Keywords:** Enzyme Inhibition, Enzyme Mechanism, Isocitrate Dehydrogenase, Kinetics, Nanoconfinement

## Abstract

The ability to control enzyme cascades entrapped in a nanoporous electrode material (the “Electrochemical Leaf”, e‐Leaf) has been exploited to gain detailed kinetic insight into the mechanism of an anti‐cancer drug. Ivosidenib, used to treat acute myeloid leukemia, acts on a common cancer‐linked variant of isocitrate dehydrogenase 1 (IDH1 R132H) inhibiting its “gain‐of‐function” activity—the undesired reduction of 2‐oxoglutarate (2OG) to the oncometabolite 2‐hydroxyglutarate (2HG). The e‐Leaf quantifies the kinetics of IDH1 R132H inhibition across a wide and continuous range of conditions, efficiently revealing factors underlying the inhibitor residence time. Selective inhibition of IDH1 R132H by Ivosidenib and another inhibitor, Novartis 224, is readily resolved as a two‐stage process whereby initial rapid non‐inhibitory binding is followed by a slower step to give the inhibitory complex. These kinetic features are likely present in other allosteric inhibitors of IDH1/2. Such details, essential for understanding inhibition mechanisms, are not readily resolved in conventional steady‐state kinetics or by techniques that rely only on measuring binding. Extending the new method and analytical framework presented here to other enzyme systems will be straightforward and should rapidly reveal insight that is difficult or often impossible to obtain using other methods.

## Graphical abstract

Transducing the tightly‐channeled catalytic activity of “nanoconfined” enzyme cascades into electrical current affords a powerful and novel way to observe specific enzyme reactions, directly and in real time. Here, the “Electrochemical Leaf” is used to extract detailed kinetic and mechanistic information on the inhibitory action of a high‐profile cancer drug that inhibits a common cancer‐associated variant of isocitrate dehydrogenase 1 (IDH1).

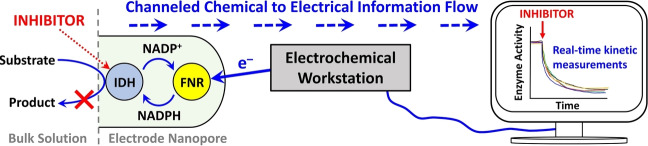

## Introduction

**Figure 1:**
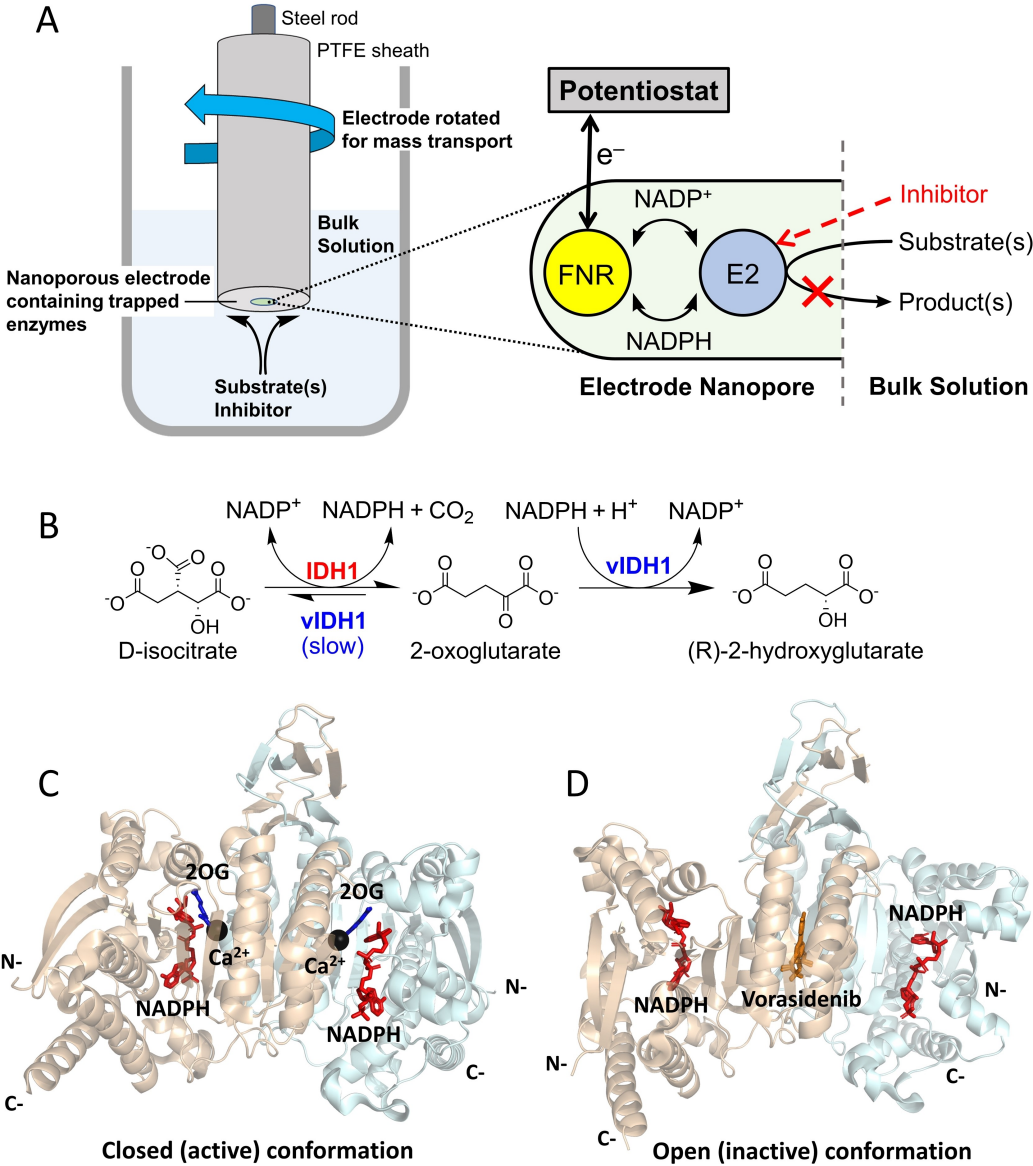
The e‐Leaf can be used to study dehydrogenase catalysis. (A) Setup of the e‐Leaf when used as an analytical tool. E2 is an NADP(H)‐dependent dehydrogenase. In this case, a rotating‐disc electrode is used to optimise substrate/inhibitor mass transport. The expanded inset shows a cartoon indicating the sequence of tightly‐channeled electrochemical information flow. (B) Reactions of wildtype IDH1 and 2HG‐producing IDH1 variants (vIDH1). Note that the variant IDH1 enzymes catalyse the wildtype reaction (isocitrate oxidation), but at a reduced rate.[^12^, ^13^, ^14^] (C and D) Views from crystal structures of IDH1 R132H. (C) The proposed closed (active) conformation with 2OG, NADP(H), and inhibitory Ca^2+^ (substituting for Mg^2+^) bound at the active site of each monomer (PDB: 4KZO).^[15]^ (D) The proposed open (inactive) conformation with one molecule of an inhibitor, Vorasidenib, bound at the dimer interface and a molecule of NADP(H) bound at the active site of each monomer (PDB: 6ADG).^[16]^

A new electrochemical platform enables the action of NAD(P)(H)‐dependent enzyme cascades to be simultaneously energized, controlled and observed in real time, under nanoconfined conditions.[^1^, ^2^, ^3^, ^4^, ^5^] Instead of being dispersed in solution, as is typical for kinetic studies, enzymes are loaded into a nanoporous material—a thin, electrically‐conductive layer of material (indium tin oxide (ITO)) formed by electrophoretic deposition of ITO nanoparticles (<50 nm) onto a conductive support (Figure 1A).^[2]^ A key element of the platform is the photosynthetic enzyme known as ferredoxin‐NADP^+^ reductase (FNR), which interacts directly with the surface of the interior nanoparticles. Facile electron tunnelling between ITO and the flavin adenine dinucleotide (FAD)‐containing active site of FNR enables rapid and reversible electrocatalytic recycling of NADP(H).[^1^, ^6^, ^7^] Largely confined within the electrode nanopores, NADP(H) can be rapidly shuttled between FNR (E1) and another NAD(P)(H)‐dependent dehydrogenase (E2). In the presence of an E2 substrate, the recycling produces an electrical current directly proportional to the activity of E2, and the tight channelling effectively renders E2 “electroactive”. The electrode potential can thus be used to drive processes in either direction, stopping or accelerating with a response time that is effectively immediate.^[2]^ Further enzymes can be added to construct extended cascades.^[3]^ The analogy with downstream photosynthesis, in which FNR plays a central role, has led to the platform being termed the “Electrochemical Leaf” (e‐Leaf).

The interactive nanoconfinement introduced by the e‐Leaf has two important consequences relevant to the study of enzyme mechanisms. Firstly, cascades that include NAD(P)(H)‐dependent enzymes can be studied at high *local* enzyme concentrations (in the millimolar range),^[8]^ a condition that in some cases may be more representative of the environment in living cells than the typically dilute solutions (often nanomolar) used for traditional enzyme kinetic assays. Essential co‐substrates can be supplied in situ,[^2^, ^3^, ^9^] and the tiny amount of enzyme present in the thin nanoporous ITO layer makes it possible to sustain a steady state (and pseudo first‐order conditions) even with low nanomolar concentrations of reactants in the surrounding bulk solution. Secondly, the catalytic rate (electrical current) is a *direct* observable, enabling the *rate of change of rate* to be readily measured with ease and precision, so informing on temporal details of inhibition processes. We envisaged that these attributes of the e‐Leaf would render it useful for characterising the kinetics of slow dehydrogenase‐inhibitor interactions, helping to resolve the events occurring in a drug‐enzyme interaction of growing importance in cancer treatment.

Human cells express three isocitrate dehydrogenases (IDHs): IDH1 is localised in the cytoplasm and IDH2 and IDH3 are localised in mitochondria.^[10]^ The IDH1/2‐encoding genes are the most commonly mutated metabolic genes that are associated with cancer.[^10^, ^11^] Both IDH1 and IDH2 are homodimers that catalyse the NADP^+^‐dependent reversible oxidative decarboxylation of isocitrate to give 2‐oxoglutarate (2OG) and carbon dioxide (Figure 1B). Active‐site mutations in IDH1 and IDH2 result in “gain of function” activity, wherein the variant enzymes preferentially catalyse the reduction of 2OG to 2‐hydroxyglutarate (2HG) (Figure 1B) with a concomitant decrease in isocitrate oxidation activity.[^12^, ^13^, ^14^]

In the case of IDH1, the focus of this study, substitution of arginine‐132 by histidine or cysteine is common (IDH1 R132H and R132C, respectively)^[14]^ in numerous cancers.[^17^, ^18^, ^19^] Small‐molecule inhibitors selective for these variants are thus under development,[^10^, ^20^, ^21^] with one drug, Ivosidenib (AG‐120), being clinically used to treat acute myeloid leukemia (AML).^[22]^ There is evidence that Ivosidenib and related molecules bind at the IDH1 dimer interface in a manner that stabilises an open, inactive conformation, i.e. they are allosteric inhibitors (Figure 1C, D).[^13^, ^14^, ^23^, ^24^, ^25^, ^26^, ^27^, ^28^] Interestingly, although IDH1 variant inhibitors bind to both wildtype IDH1 and IDH1 variants, in many cases only the variant is inhibited.[^13^, ^29^]

Comparisons of enzyme inhibitor potency are often based on *K*_i_ or IC_50_ values (the latter being the concentration required to reduce activity by 50 % under specific conditions). These values do not inform on the timescale or mechanism of inhibition (which may involve several steps) or how inhibition alters in response to different turnover conditions, i.e., when the enzyme is catalytically active and present in different conformations that may affect drug binding. Detailed kinetic data are frequently omitted (for logistical/cost reasons) in initial screening for inhibitors,[^30^, ^31^, ^32^, ^33^] despite the importance of understanding kinetic parameters such as the association rate constant, *k*_on_, dissociation rate constant, *k*_off_, residence time, *t*_r_=1/*k*_off_ and half‐life, *t*_1/2_=ln(2)/*k*_off_, for establishing the mechanism and optimising in vivo efficacy. Such kinetic data, measured under conditions that allow enzyme turnover, are particularly important for analysing slow‐acting inhibitors, such as Ivosidenib,^[30]^ and conformationally dynamic enzymes such as the IDHs.^[34]^

The limitations of standard methods applied for initial inhibitor screens do not apply to the e‐Leaf, which provides direct kinetic data, including for slow‐acting inhibitors, under turnover conditions. Using the inhibition of IDH1 R132H by Ivosidenib as a case study, we demonstrate here how the e‐Leaf can provide a breadth of mechanistically crucial kinetic information that is efficiently acquired yet rich in detail. Given the potential of the e‐Leaf to be miniaturised, it has the capacity to provide high‐throughput information on the kinetics of inhibition.

## Results and Discussion

### Dual enzyme monitoring in an electrode nanoreactor—a vivid demonstration of selective inhibition

The e‐Leaf enables two different reactions (oxidation and reduction), catalysed by separate enzymes, to be compared simultaneously in a single experiment using cyclic voltammetry, providing insight that is not otherwise directly attainable. We compared the actions of Ivosidenib on wildtype IDH1 (which catalyses isocitrate oxidation) and IDH1 R132H (which catalyses 2OG reduction), aiming to mimic the selectivity of inhibition as it might be manifest in a cancer patient, where both wildtype and variant IDH1 are present.

**Figure 2:**
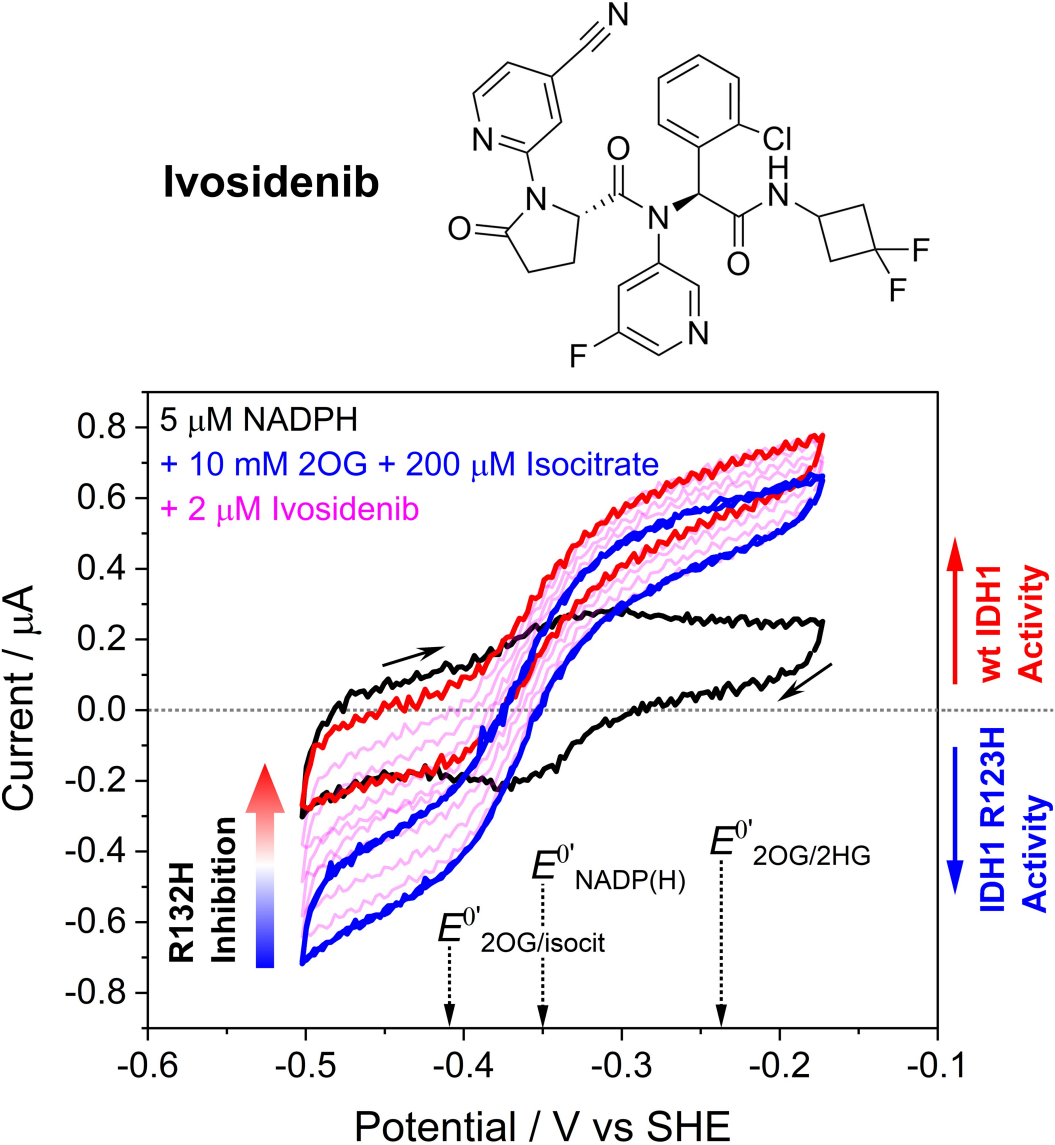
Dual enzyme monitoring demonstrates inhibitor selectivity. Top. Structure of Ivosidenib. Bottom. Cyclic voltammograms showing simultaneous effects on the activities of wildtype IDH1 and IDH1 R132H after introducing Ivosidenib, which selectively inhibits IDH1 R132H. Black trace: 5 μM NADPH (without isocitrate or 2OG present). Blue traces: two consecutively obtained traces overlaid (to show the system is stable) with 10 mM 2OG and 0.2 mM enantiopure *D*‐isocitrate. The pink traces were obtained after injecting Ivosidenib (2 μM). Note that the oxidation current is solely due to wildtype IDH1 isocitrate oxidation activity (the small amount of IDH1 R132H isocitrate oxidation activity is not measured when 2OG is present)[^2^, ^14^] and the reduction current is solely due to IDH1 R132H 2OG reduction activity (wildtype IDH1 requires high [CO_2_] to reduce 2OG to isocitrate).^[2]^ See Figure S1 for control experiments. Conditions: (FNR+IDH1_WT_+IDH1_R132H_)@ITO/PGE electrode, electrode area 0.06 cm^2^, electrode rotation rate 1000 rpm, scan rate 1 mV/s, temperature 25 °C, O_2_<1 ppm, volume 4 mL, pH=8 (100 mM HEPES), 10 mM MgCl_2_, 5 μM NADPH, enzyme loading ratios (molar): FNR/IDH1_WT_/IDH1_R132H_; 2/1/5.

Experiments in which the electrode was loaded with both wildtype IDH1 and IDH1 R132H reveal the selective action of Ivosidenib on R132H catalysis while leaving wildtype IDH1 activity unaffected (Figure 2). An initial voltammogram (black trace) recorded with a cell solution containing 5 μM NADPH without either IDH substrate present shows the background FNR‐catalysed conversion of nanoconfined NADP^+^ into NADPH (reduction peak) and NADPH into NADP^+^ (oxidation peak), in the presence of IDH1 and IDH1 R132H. Isocitrate and 2OG, the substrates of wildtype IDH1 and IDH1 R132H, respectively, were then added and two consecutive voltammograms recorded (blue traces) to confirm activities and stability. Ivosidenib was then added and successive voltammograms (pink traces) recorded to monitor changes in wildtype and R132H IDH1 activity with time. The results reveal the highly selective Ivosidenib‐mediated inhibition of IDH1 R132H‐catalysed 2OG reduction (at a potential negative with respect to the NADP^+^ reduction peak). Isocitrate oxidation catalysed by wildtype IDH1 (at a potential positive of the NADPH oxidation peak) is not inhibited: the final voltammogram (red trace) shows a slight increase in oxidation current, which can be attributed to the removal of the pathway (R132H‐catalysed reduction of 2OG) that competed with the NADPH oxidation catalysed by FNR.

### Obtaining pseudo first‐order kinetics at nanomolar inhibitor concentrations

**Figure 3:**
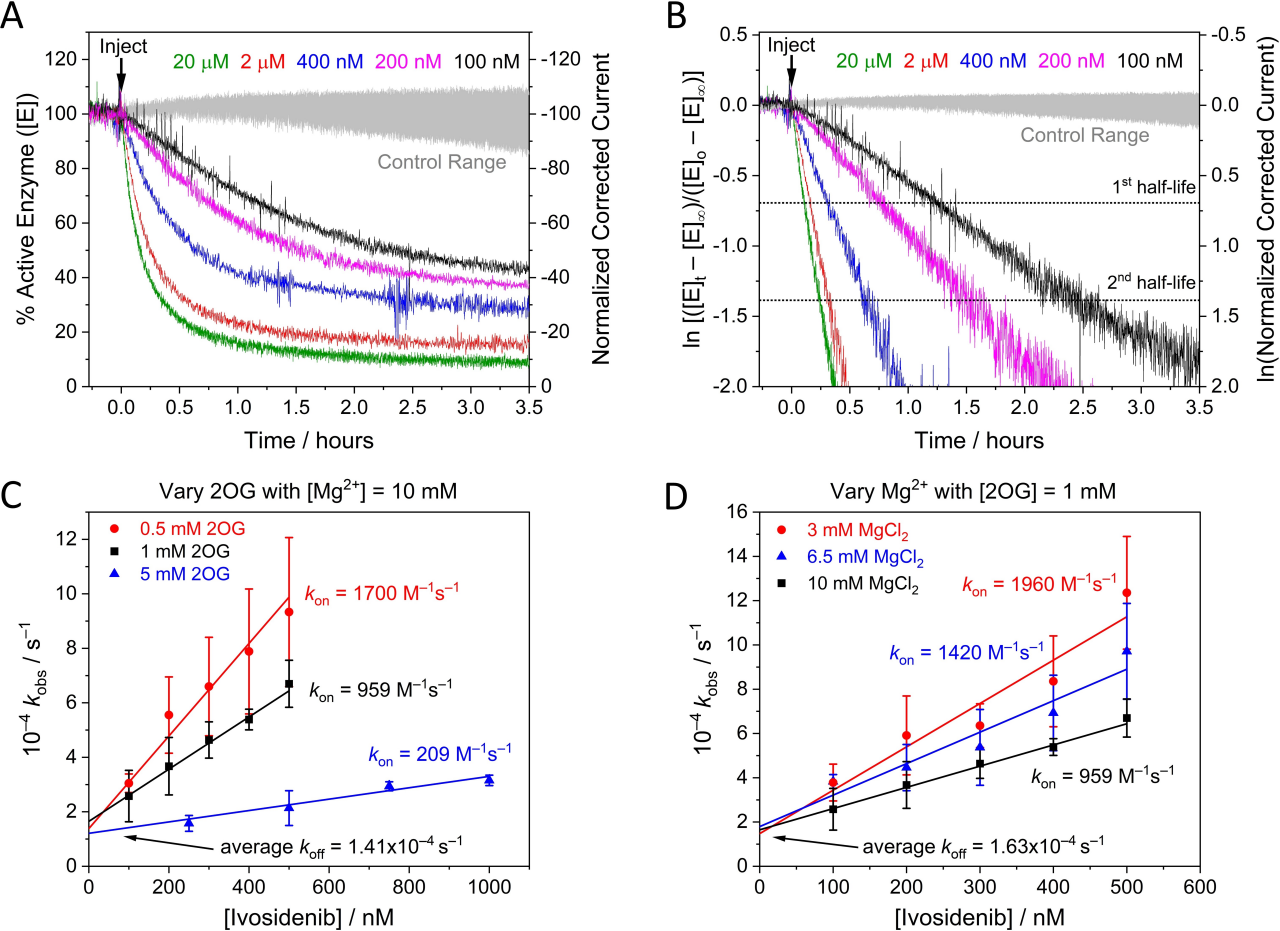
Kinetic plots for IDH1 R132H inhibition at different Ivosidenib, 2OG, and Mg^2+^ concentrations (A) Decrease in the amount of active IDH1 R132H versus time (using enzyme rate/current as a proxy, see Supporting Information) following injection of different Ivosidenib concentrations (0.1–20 μM). The data in panel A were corrected for film loss (see Materials and Methods); uncorrected data are presented in Figure S2A. (B) Data from panel A plotted logarithmically (Eq. S11) showing that inhibition exhibits pseudo first‐order reaction kinetics for over two half‐lives. The equilibrium value for each data set was obtained from the film loss corrected plot (panel A). (C and D) Observed rate constant (*k*_obs_) values for IDH1 R132H inhibition at different Ivosidenib concentrations and with varied concentrations of 2OG (C) and MgCl_2_ (D). The slope=*k*_on_, the intercept=*k*_off_, and intercept/slope=*K*_d_ (see Eq. 2). Conditions: (FNR+IDH1_R132H_)@ITO/PGE electrode, electrode area 0.06 cm^2^, electrode rotation rate 1000 rpm, temperature 25 °C, *E*=−0.5 V vs SHE, O_2_<1 ppm, volume 4 mL, pH=8 (20 mM each of: MES, TAPS, CHES), 10 μM NADPH, enzyme loading ratios (molar): FNR/IDH1_R132H_; 1/2.5. The data in panel C were measured at MgCl_2_=10 mM; the data in panel D were measured at 2OG=1 mM. Note: data in black in panels C and D are the same.

Timecourses for inhibition of IDH1 R132H by Ivosidenib were investigated in detail by monitoring changes in the rate of 2OG reduction with the electrode potential maintained at −0.5 V. Electrode rotation drives mass transport of both the substrate and inhibitor to and from the ITO layer in which the enzymes are loaded (see Figure 1A). The results (Figure 3A) show that injections of Ivosidenib rapidly initiate decreases in catalytic activity, the rates and extents of which increase with Ivosidenib concentration, with the lowest tested concentration (100 nM) achieving <75 % inhibition after 3.5 h. The results clearly imply that a limiting rate is approached at high Ivosidenib concentration (it will be shown later that the kinetics are not limited by nanoconfinement). The same data plotted in semi‐log form (Figure 3B **)** show that all the reactions exhibit pseudo first‐order kinetics (see Supporting Information) for at least two half‐lives, i.e. the rate is proportional to the amount of enzyme active at the time that has elapsed. Note the very large range of inhibitor concentrations, ranging from nanomolar to micromolar levels and above, that can be studied continuously in a uniform e‐Leaf procedure. The ability to analyse low inhibitor levels in this straightforward way is possible because, although the local concentration of IDH1 R132H in the nanopores is high, the total amount present (around 17 pmoles)^[8]^ is much less than the total amount of inhibitor in the bulk solution “reservoir” (0.4–80 nmoles in these experiments). Transport of small molecules within the ITO layer is not a limiting factor except for very fast reactions.^[35]^ The inhibitor concentration thus remains effectively constant throughout the measurement and pseudo first‐order conditions apply. Importantly, the kinetic traces represent the *transient* kinetics of inhibition during *steady‐state* catalysis (i.e. the rate of change of catalytic rate). Henceforth, these high‐quality and reproducible primary data were amenable to analysis and interpretation using familiar “textbook” kinetics procedures.

### Extracting k_on_, k_off_, and K_d_ values

The results were used to investigate how the pseudo first‐order rate constant, *k*_obs_, varies with relatively low concentrations of Ivosidenib (<1 μM) for three different concentrations of 2OG and Mg^2+^ (Figure 3C and D, respectively); Mg^2+^ is required for the productive binding of 2OG.^[13]^ The gradients of the lines vary with the 2OG or Mg^2+^ concentration, but the plots meet at a common intercept. Non‐denaturing mass spectrometry reveals that Ivosidenib binds to wildtype IDH1 and IDH1 R132H with a stiochoimetry of one pwr IDH1 dimer.^[29]^ Therefore, consistent with the results in Figure 3A, which shows that inhibition is incomplete and reaches or approaches an equilibrium value, the results imply a reversible reaction between enzyme, E, and a single molecule of inhibitor, I, of the type:

(1) $$E+I\overset{k_{\mathrm{on}}}{\underset{k_{o\mathrm{ff}}}{↔}}\mathrm{EI}$$

for which the observed rate constant, *k*_obs_, (see Eqs. S11, S12) is:

(2) $$k_{\mathrm{obs}}=k_{\mathrm{on}}\left[I\right]+k_{\mathrm{off}}$$

The gradient corresponds to the second‐order rate constant for the inhibition reaction (*k*_on_) while the intercept gives the first‐order rate constant for its reversal (*k*_off_). The microscopic inhibitory equilibrium constant ($K_{d}^{I}$ ) for Ivosidenib is given by the ratio of the intercept to the gradient (*k*_off_/*k*_on_).

### How inhibition of IDH1 R132H depends on levels of 2OG and Mg^2+^

The common intercept (*k*_off_) with variation of both 2OG and Mg^2+^ has an average value (across the 5 data sets presented in Figure 3C, D) of 1.50×10^−4^ s^−1^, indicating that the rate at which inhibition by Ivosidenib is reversed to restore active enzyme is *independent* of the tested 2OG or Mg^2+^ concentrations. By contrast, the fact that *k*_on_ values decrease as the concentrations of 2OG or Mg^2+^ are raised suggests that Ivosidenib acts as an inhibitor only when either or both 2OG or Mg^2+^ are not bound, consistent with studies using standard methods.[^13^, ^20^] The data were used to derive an outline mechanism, wherein it was assumed, for simplicity, that the binding of a single molecule or ion X (2OG or Mg^2+^) with dissociation constant $K_{d}^{X}$ prevents the enzyme from being inhibited by Ivosidenib. The reactions are:
(3)

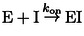



(4)

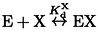




from which it follows that (see Supporting Information)

(5) $$k_{\mathrm{on}}=\frac{k_{\mathrm{on}}^{0}K_{d}^{X}}{K_{d}^{X}+\left[X\right]}$$

where $k_{\mathrm{on}}^{0}$ is the second‐order rate constant at [X]=0. Taking reciprocals gives

(6) $$\frac{1}{k_{\mathrm{on}}}=\frac{1}{k_{\mathrm{on}}^{0}}+\frac{\left[X\right]}{k_{\mathrm{on}}^{0}K_{d}^{X}}$$

**Figure 4:**
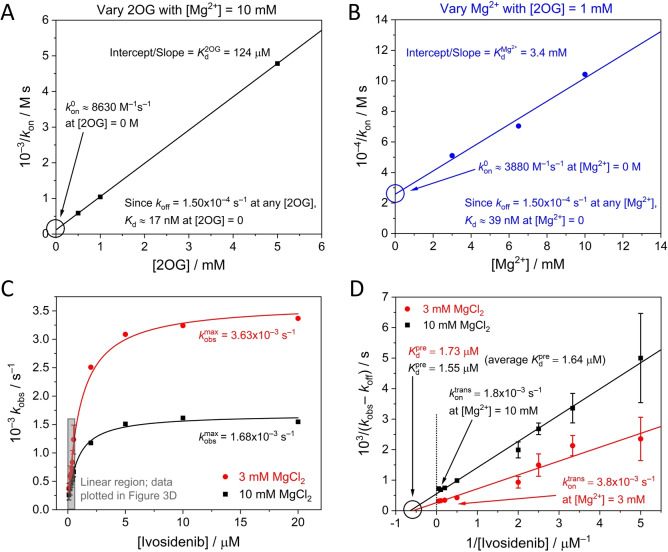
(A and B) *k*_on_ data from the lines of best fit in Figure 3C and D plotted using Eq. 6 to obtain limiting Ivosidenib $k_{\mathrm{on}}^{0}$ values (and *K*_d_ values) where [2OG] and [Mg^2+^] equal 0 M. (C) Plot showing how *k*_obs_ varies with the Ivosidenib concentration at two concentrations of Mg^2+^ ([2OG]=1 mM). The rate of inhibition is initially linear with increasing inhibitor concentrations but ultimately reaches a limiting value that is Mg^2+^ dependent. The grey rectangle re‐shows the data from Figure 3D. The *k*_obs_ values outside of the linear region were measured under the same experimental conditions as those described in Figure 3D (2OG=1 mM); each of these data points represents a single experiment. (D) Data from Figure 4C plotted according to Eq. 11 to obtain $k_{\mathrm{on}}^{\mathrm{trans}}$ and $K_{d}^{\mathrm{pre}}$ values.

As shown in Eq. 6, plots of 1/*k*_on_ vs [2OG] or [Mg^2+^] should be linear, with slope=$1/k_{\mathrm{on}}^{0}K_{d}^{X}$ , and intercept=$1/k_{\mathrm{on}}^{0}$ . The dissociation constants $K_{d}^{X}$ for 2OG (at a given Mg^2+^concentration) or for Mg^2+^ (at a given 2OG concentration) are given by the ratio intercept/gradient (Figure 4A, B). The values obtained ($K_{d}^{2\mathrm{OG}}$ =0.124 mM; $K_{d}^{{\mathrm{Mg}}^{2+}}$ =3.4 mM) are consistent with corresponding Michaelis constants (*K*_m_) determined using standard solution‐based spectrophotometric assays ($K_{m}^{2\mathrm{OG}}$ =0.40 mM; $K_{m}^{{\mathrm{Mg}}^{2+}}$ =4 mM for Mg^2+^).^[14]^

The results enable the inhibitory action of Ivosidenib on IDH1 R132H to be predicted over a range of conditions assuming that the inhibitory equilibrium constant is represented by the kinetic relationship $K_{d}^{I}=k_{\mathrm{off}}/k_{\mathrm{on}}$ within the sub‐micromolar range. Thus at [2OG]=0 (the condition for which $K_{d}^{I}=k_{\mathrm{off}}/k_{\mathrm{on}}^{0}$ ), $K_{d}^{I}=$ 17 nM; similarly, at [Mg^2+^]=0, $K_{d}^{I}=$ 39 nM. These data are in broad agreement with IC_50_ values determined after enzyme‐inhibitor pre‐incubation (performed without either 2OG or Mg^2+^ present).[^10^, ^14^, ^20^, ^36^] The inhibition of R132H by Ivosidenib is weakened as the 2OG or Mg^2+^ concentrations are increased to more likely physiologically‐relevant levels; thus, for the condition [2OG]=1 mM and [Mg^2+^]=10 mM, $K_{d}^{I}=$ 153 nM. Ivosidenib inhibition appears more sensitive to changes in 2OG compared to Mg^2+^ concentrations, an observation consistent with 2OG having a much lower *K*_d_ than Mg^2+^; however, Mg^2+^ is required for 2OG to bind the enzyme efficiently,^[13]^ and since the $K_{d}^{2\mathrm{OG}}$ value (and all steady‐state *K*_m_ values) was measured with Mg^2+^ present (both 2OG and Mg^2+^ are required for turnover), the effects of 2OG and Mg^2+^ cannot be assessed independently.

### Evidence that Ivosidenib binds rapidly before it inhibits the activity of IDH1 R132H

At high Ivosidenib concentrations (>1 μM) the rate of inhibition increases to reach a limiting value (Figure 4C). Here, *k*_obs_ values are plotted over an extended range of Ivosidenib concentrations for two Mg^2+^ concentrations: 3 and 10 mM. The results imply a hyperbolic dependence of *k*_obs_′ (observed rate constant adjusted for intercept, see Supporting Information) on higher inhibitor concentration described by the empirical equation:

(7) $$k_{\mathrm{obs}}^{'}=\frac{a\left[I\right]}{b+\left[I\right]}$$

for which we considered two options that involve initial reversible but *non‐inhibitory* binding (see Supporting Information for extended discussion): (1) inhibition of IDH1 R132H proceeds in two linear stages, an initial rapid and reversible binding step to generate a precursory enzyme‐inhibitor complex that is still active, followed by a first‐order (intramolecular) step that results in inhibition; (2) the inhibitor binds in two modes, one that inhibits, the other yielding a complex that is still fully active but resistant to inhibition. Both options yield the same empirical rate law (Eq. 7). Although we could not rule out the possibility of mechanism 2, mechanism 1 is well‐established for slow, tight binding inhibitors.[^30^, ^33^, ^37^, ^38^]

With mechanism 1, the first stage rapidly generates an initial enzyme‐inhibitor complex in a reversible process described by a pre‐equilibrium constant, *K* ^pre^, 
(8)

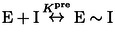




which is followed by a first‐order (intramolecular *trans*formative) step that results in inhibition.

(9) $$E∼I\overset{k_{\mathrm{on}}^{\mathrm{trans}}}{\underset{k_{\mathrm{off}}^{\mathrm{trans}}}{↔}}\mathrm{EI}$$

Noting, as before, that the latter step is also reversible, and assuming $k_{\mathrm{off}}^{\mathrm{trans}}$ =*k*_off_ (as $k_{\mathrm{off}}^{\mathrm{trans}}$ should approximate to the rate‐determining step in that direction), we adopted the approach of Morrison and Walsh^[38]^ to derive (see Supporting Information):

(10) $$k_{\mathrm{obs}}-k_{\mathrm{off}}=\frac{k_{\mathrm{on}}^{\mathrm{trans}}\left[I\right]}{K_{d}^{\mathrm{pre}}+\left[I\right]}$$

Taking reciprocals:

(11) $$\frac{1}{\left(k_{\mathrm{obs}}-k_{\mathrm{off}}\right)}=\frac{1}{k_{\mathrm{on}}^{\mathrm{trans}}}+\frac{K_{d}^{\mathrm{pre}}}{k_{\mathrm{on}}^{\mathrm{trans}}}\cdot \frac{1}{\left[I\right]}$$

Consequently, a plot of $1/\left(k_{\mathrm{obs}}-k_{\mathrm{off}}\right)$ vs 1/[I] should give a straight line with a y‐intercept of $1/k_{\mathrm{on}}^{\mathrm{trans}}$ and an x‐intercept=$-1/K_{d}^{\mathrm{pre}}$ . The results (Figure 4D) show that measurements at different Mg^2+^ concentrations converge to a common x‐intercept value, thereby indicating that the initial binding of inhibitor to the enzyme, represented by $K_{d}^{\mathrm{pre}}$ and having a measured value of 1.64 μM, is unaffected by Mg^2+^. By contrast, the data cut the y‐axis at different points, showing that the latter stage of the inhibitory process ($k_{\mathrm{on}}^{\mathrm{trans}}$ ) is Mg^2+^‐dependent, with higher Mg^2+^ concentrations slowing the reaction.

### Comparing inhibition kinetics in crowded nanopores vs dilute solution

**Figure 5:**
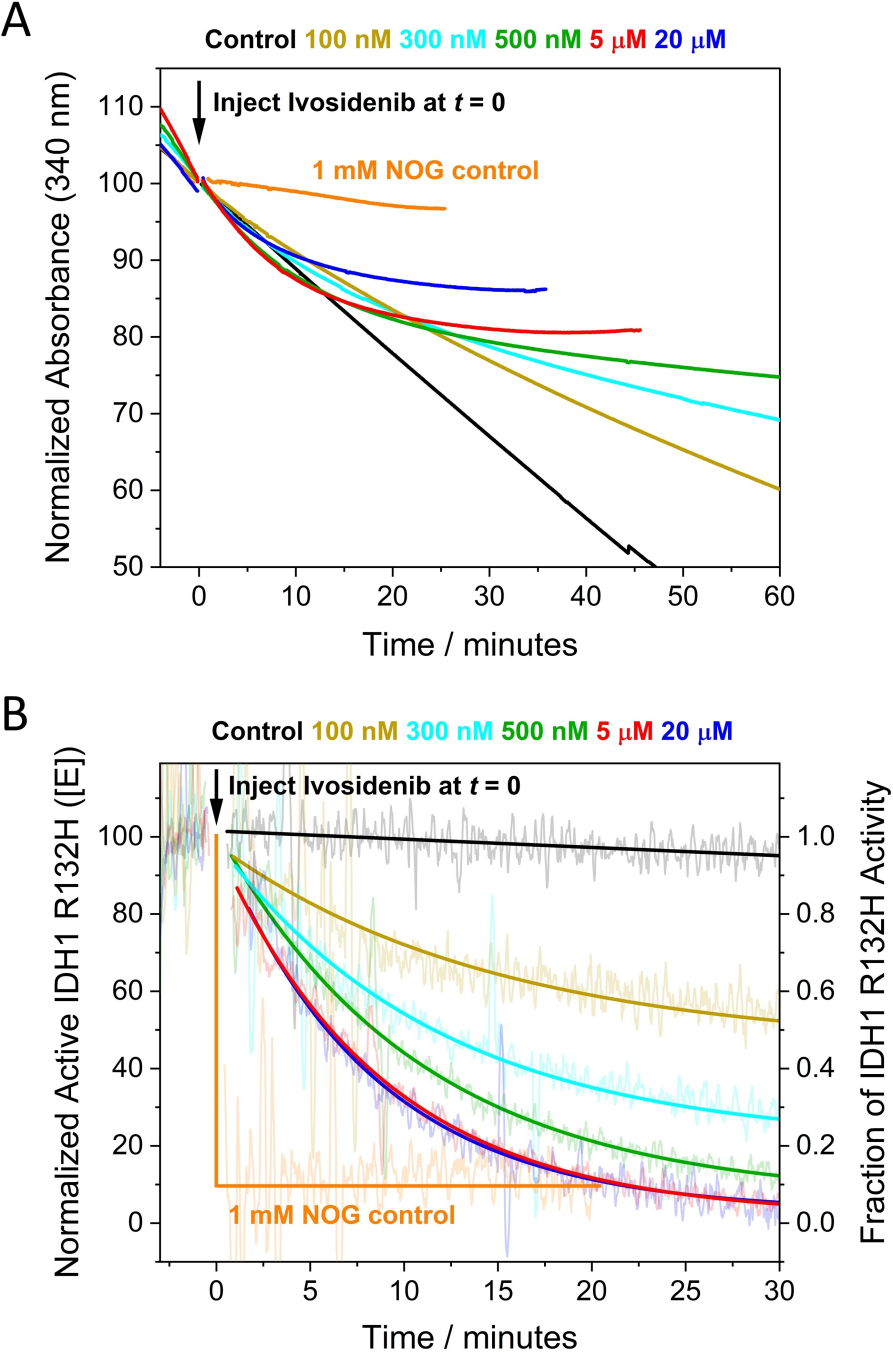
Kinetics of IDH1 R132H inhibition by Ivosidenib measured in dilute solution. (A) Normalized absorbance (at 340 nm to monitor NADPH) versus time showing how the rate is affected by different concentrations of Ivosidenib injected at *t*=0. Ivosidenib was added to the reaction as a concentrated 1 μL solution in DMSO; the control experiment used DMSO without inhibitor. (B) Data from panel A converted into derivative form (i.e., enzyme rate vs time) to measure the rate of inhibitor‐induced changes to the enzyme rate (rate of change of rate) and the extent of inhibition at different inhibitor concentrations. Note: experiments at 2 and 10 μM Ivosidenib showed roughly the same rate of inhibition as 5 and 20 μM but were omitted for clarity.

We investigated the kinetics of IDH1 R132H inhibition in dilute solution, with the aims of comparing two very different environments (dilute vs crowded enzyme) and investigating the extent to which the conventional approach can resolve the complex kinetics (Figure 5). In order to study the slow inhibition occurring during otherwise steady‐state catalysis, it was important that the rate of depletion of NADPH without inhibitor (based on absorbance at 340 nm) was constant for a substantial period: this was achievable for>30 min. using 10 mM Mg^2+^, 1 mM 2OG and a R132H solution concentration of 100 nM, which is ≈10,000‐fold less concentrated than the estimated *local* 1–2 mM R132H concentration in the electrode nanopores.^[8]^ Figure 5A shows plots of absorbance (normalized) vs time after introducing different concentrations of Ivosidenib. A control experiment was carried out with an active‐site binding inhibitor, N‐oxalylglycine (NOG), a catalytically‐inactive 2OG analogue,[^13^, ^39^] which manifested ≈90 % inhibition within seconds. The data were converted into the first derivative to obtain the *rate of change* of catalytic rate with time (Figure 5B), giving a plot analogous to Figure 3A. In agreement with electrochemical experiments, the lowest concentrations of Ivosidenib in dilute solution did not fully inhibit catalysis, and the rate of inhibition increased with inhibitor concentration, reaching a limiting value at approximately 10 μM (Figure 4C) (Figure S6).

In dilute solution, the experiments performed at the lowest Ivosidenib concentrations required to resolve *k*_on_ and *k*_off_ are not obtained under pseudo first‐order conditions, and the small changes to the initial rate are difficult to analyse reliably. At higher Ivosidenib inhibitor concentrations (2–20 μM), pseudo first‐order conditions apply, and the data were used to determine the maximum (limiting) rate of inhibition (Figure S6). The maximum observed rate constant obtained for the solution experiments, 2.0×10^−3^ s^−1^ (half‐time of 5.8 min.) is in good agreement with that derived for the analogous conditions using the e‐Leaf approach, 1.68×10^−3^ s^−1^ (half‐time of 6.9 min.). Taken together, the observations and data for inhibition in dilute solution show that nanoconfinement does not substantially affect the kinetics of inhibition (see Supporting Information for extended discussion).

### Timeline for IDH1 R132H inhibition by Ivosidenib and implications for investigations of other allosteric inhibitors

**Figure 6:**
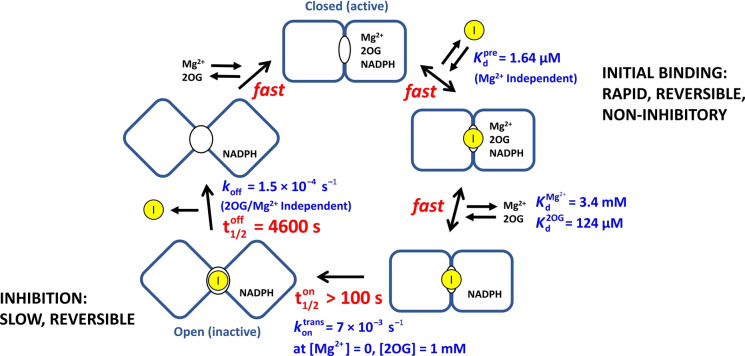
Timeline for the inhibition of IDH1 R132H by Ivosidenib showing the fast and slow steps involved. For simplicity, the binding/dissociation steps for 2OG and Mg^2+^ are shown at a single monomer active site, (though there is likely half‐site reactivity). The pre‐equilibrium dissociation constant, $K_{d}^{\mathrm{pre}}$ , was measured from data shown in Figure 4D. $K_{d}^{2\mathrm{OG}}$ and $K_{d}^{{\mathrm{Mg}}^{2+}}$ were determined from data shown in Figure 4 panels A and B, respectively. For the subsequent slow steps, the t_1/2_ values are first‐order complex half‐times calculated using the rate constants given in blue for each step. The $k_{\mathrm{on}}^{\mathrm{trans}}$ value presented is the limiting (fastest) rate, which was determined by plotting the Mg^2+^‐dependent $k_{\mathrm{on}}^{\mathrm{trans}}$ values measured in Figure 4D against [Mg^2+^] and extrapolating to [Mg^2+^]=0 M.

The results enable a timeline to be drawn up for inhibition of IDH1 R132H by Ivosidenib (Figure 6). The process occurs in two stages: initial rapid (non‐inhibitory) binding to the active enzyme, $K_{d}^{\mathrm{pre}}$ , followed by an intramolecular step with rate constant $k_{\mathrm{on}}^{\mathrm{trans}}$ that results in inhibition. Importantly, *reversal* of the latter step is a slow process with a microscopic first‐order rate constant (*k*_off_) that is independent of the presence of 2OG or Mg^2+^. The data thus yield a half‐life of 4600 seconds, corresponding to an intrinsic residence time (1/*k*_off_) of 6600 seconds at 25 °C (just under two hours).

## Conclusion

As demonstrated by studies on IDH1 variant inhibition by the pioneering cancer drug, Ivosidenib, the results reveal how the e‐Leaf efficiently provides new and detailed mechanistic insight. The results are likely to have general relevance for IDH variant inhibition: a parallel, but less exhaustive, investigation carried out with another allosteric inhibitor of IDH1 R132H, Nov224, revealed behavior similar to that observed with Ivosidenib, i.e., two‐step binding (competitive with Mg^2+^/2OG) and a comparable inherent drug residence time, but with small differences in rates (Figures S3–S5 and Table S1).

Despite Ivosidenib being used clinically, no detailed kinetic information on its mechanism is available, and an information gap separates empirical measurements of efficacy or binding and the actual kinetics that underlie its mechanism of action. Literature reports on Ivosidenib and other drug candidates have focused on IC_50_ and related quantities,[^14^, ^20^, ^36^] or direct binding measurements under non‐turnover conditions employing surface plasmon resonance (SPR), non‐denaturing mass spectrometry, NMR, and isothermal titration calorimetry.[^13^, ^14^, ^29^, ^40^] The reason for the shortage of kinetic information is that Ivosidenib has an IC_50_ that is below enzyme concentrations that are experimentally practical for studying the slow inhibition kinetics. Importantly, the values measured using the e‐Leaf are consistent with kinetic studies on other IDH inhibitors[^23^, ^25^, ^40^] (Table S1), but provide further detailed information that is not readily obtained using standard methods, and which may be more reliable. For example, evidence compiled in a recent report^[29]^ showed that allosteric IDH inhibitors bind tightly to both the wildtype *and* variant IDH enzymes with roughly equal affinity despite only the variant enzymes being inhibited. The clear implication of such a lack of correlation is that the results of binding‐only assays can lead to incorrect conclusions. By contrast, a single experiment using the e‐Leaf highlights inhibitor selectivity (Figure 2) and extended data reveal the presence of more than one inhibitor‐enzyme binding mode for the IDH1 R132H variant (tight inhibitory and weaker non‐inhibitory). Together, these results clarify how a potential drug can bind tightly (at the nano‐to‐micromolar level) yet *not* be an inhibitor and emphasize why equilibrium or kinetic studies based on binding alone can be misleading. The results also suggest an issue that could arise when two drugs are applied in parallel, i.e. combination therapy, which was recently suggested for IDH inhibitors;^[41]^ if a common two‐step linear pathway is operative, the less potent inhibitor may bind the enzyme in a non‐inhibitory conformation, blocking the more potent inhibitor, thus resulting in weaker overall enzyme inhibition.

Crucially, the fastest rates of inhibition observed for IDH1 enzymes concentrated to millimolar levels in the ITO nanopores^[8]^ can be reproduced in homogeneous solution (100 nM enzyme, Figure 5). Such an observation implies that the mass transport of inhibitor in the bulk solution to enzyme molecules deeply buried in the electrode is not rate‐limiting. This proposal is supported by a recent computational study^[35]^ which concluded that small molecules are able to permeate the ITO layer rapidly and homogeneously, i.e., diffusion *within* the electrode should not be rate‐limiting when the reaction of interest is sufficiently slow (see Supporting Information for extended discussion). These results thus provide compelling evidence that, for this example at least, the inherent reactivity of enzyme molecules that are highly concentrated in a nanoconfined environment may not differ significantly from that observed in very dilute solution. Unlike homogeneous solution conditions, however, an important property of the heterogeneous e‐Leaf platform is its ability to sustain pseudo first‐order conditions at very low inhibitor concentration, since the total amount of inhibitor present in the bulk solution may still be much greater than the total amount of enzyme under study. This feature simplifies the interpretation of results and enables experiments over a wide concentration range of inhibitor and substrate/cofactor (nanomolar to millimolar and beyond) under conditions of enzyme turnover, yielding *both* kinetic and equilibrium information. By contrast, procedures for the most ubiquitous measurement of IDH inhibition, i.e. an IC_50_ value, typically involve pre‐incubation of potential inhibitors, which thus, at least initially, bind under non‐turnover conditions.[^25^, ^36^] Depending on the rate at which the inhibitor acts, the length of the inhibitor‐enzyme incubation time (literature values range from 12 min^[14]^ to 30 min[^20^, ^40^] to 16 h.[^25^, ^36^]) can affect the IC_50_ value:^[26]^ consequently, information on the mechanism under turnover conditions can be obscured.

Overall, the e‐Leaf has three major advantages as a platform for investigating inhibitor‐enzyme interactions over more conventional methods (e.g. analysing binding by surface plasmon resonance (SPR), NMR or MS and initial enzyme turnover rate measurements): (1) the e‐Leaf enables efficient and comprehensive quantitative kinetic investigations over a continuous concentration range spanning many orders of magnitude – a “panoptic” (wide‐angle) view of the kinetic landscape being obtained with a single method. Importantly, pseudo first‐order conditions apply down to the nanomolar potency level that is relevant for the most effective drugs, thereby enabling information to be obtained for the full duration of a process; (2) the measurement is activity based, meaning accurate rates of inhibition and its reversal are measured; in contrast, binding‐only methods are not (as tacitly assumed) necessarily a reliable indicator of enzyme inhibition;[^13^, ^29^] (3) the ability to control and measure, simultaneously, the activities of multiple enzymes “side‐by‐side” in the same nanoconfined environment, as clearly demonstrated for Ivosidenib targeting R132H while leaving the wildtype IDH1 unscathed (Figure 2), is unique, opening up new possibilities for studying enzyme‐small molecule and enzyme‐enzyme interactions as well as myriad other processes that can be coupled to the activity of a dehydrogenase‐FNR pair (Figure 1). The e‐Leaf is a transformative technology, and to make it industrially‐relevant, the next step will be to miniaturize and multiplex the technology so that it is capable of performing a host of separate reactions in parallel for high‐throughput applications.

## Supporting Information

The authors have cited additional references within the Supporting Information.[^42^, ^43^, ^44^, ^45^]

## Conflict of interest

The authors declare no conflict of interest.

1

## Supporting information

As a service to our authors and readers, this journal provides supporting information supplied by the authors. Such materials are peer reviewed and may be re‐organized for online delivery, but are not copy‐edited or typeset. Technical support issues arising from supporting information (other than missing files) should be addressed to the authors.

Supporting Information

## Data Availability

The data that support the findings of this study are available in the supplementary material of this article.
