# Supplementary material for: Electrochemical Nanoreactor Provides a Comprehensive View of Isocitrate Dehydrogenase Cancer‐drug Kinetics
Source: Angew Chem Weinheim Bergstr Ger. 2023 Sep 12;135(42):e202309149. doi: 10.1002/ange.202309149 (PMC10962547; doi:10.1002/ange.202309149)
Supplement: Supplementary file 1 — Supporting Information [file ANGE-135-0-s001.pdf]

Angewandte Chemie
Eine Zeitschrift der Gesellschaft Deutscher Chemiker

Supporting Information

Electrochemical Nanoreactor Provides a Comprehensive View of Isocitrate Dehydrogenase Cancer-drug Kinetics

*R. A. Herold, C. J. Schofield, F. A. Armstrong**

Supporting Information

Electrochemical Nanoreactor Provides a Comprehensive View of Isocitrate Dehydrogenase Cancer-drug Kinetics

Ryan A. Herold[1], Christopher J. Schofield[2], and Fraser A. Armstrong[1]

[1]Inorganic Chemistry Laboratory, Department of Chemistry, University of Oxford, Oxford OX1 3QR, United Kingdom.

[2]Department of Chemistry and the Ineos Oxford Institute for Antimicrobial Research, University of Oxford, Oxford OX1 3QY, United Kingdom.

Materials and Methods

Chemicals and reagents

Reagents used: $NADP^+$ (monosodium salt, 98%, Melford), NADPH (tetrasodium salt, 93%, Melford), acetone (ACS Reagent), indium tin oxide (ITO) powder (< 50 nm particle size, Sigma-Aldrich), (HEPES) (free acid, Melford), [2-(N-morpholino)-ethanesulfonic acid] (MES) (monohydrate, Melford), [tris(hydroxymethyl)methylamino]-propanesulfonic acid (TAPS) (99%, Melford), [2-(N-cyclohexylamino)ethanesulfonic acid] (CHES) (>99%, Melford), [N-(2-hydroxyethyl) piperazine N'-(2-ethanesulfonic acid)], α-ketoglutaric acid (disodium salt dihydrate, ≥98%, Sigma-Aldrich), (+)-potassium Ds-threo-isocitrate (monobasic, ≥98%, Sigma-Aldrich), $MgCl_2$ (anhydrous, Melford), iodine (resublimed crystals, 99.9985% (metals basis), puratronic, Alfa Aesar), isopropyl β-D-1-thiogalactopyranoside (IPTG) (Alfa Aesar). Aqueous solutions were made using ultrapure water (≥18.2 MΩ cm, Milli-Q). Inhibitors: Ivosidenib (AG-120) (Cat. No.: HY-18767, 99.78%) and Nov224 (Cat. No.: HY-18717, >98.0%) were obtained from MedChemExpress and were dissolved in DMSO (10 mM) and stored at -80 °C.

Enzyme expression and purification

Recombinant human isocitrate dehydrogenase 1 (wildtype and the R132H variant) and ferredoxin $NADP^+$-reductase (FNR) from *Chlamydomonas reinhardtii* were expressed and purified as reported.[1,2]

Electrodes

Nanoporous indium tin oxide (ITO) electrodes were made by electrophoretic deposition of ITO nanoparticles (<50 nm, Sigma-Aldrich) onto pyrolytic graphite edge (PGE) rotating disc electrodes as previously described,[1] except that the deposition time was increased to 8–12 minutes to produce a thicker ITO layer. In-house rotating disc electrodes (PGE) were prepared as reported.[3] ITO nanoparticles (20 mg) and I_2 (10 mg) were added to 20 mL of acetone and the mixture was sonicated for 1–2 hours. The PGE electrode and a counter electrode (ITO on glass) were held in a parallel orientation approximately 1 cm apart (with the PGE electrode facing down) in the ITO suspension with stirring provided by a magnetic stir

bar. A voltage of 10 V was applied (zero current) for 6 minutes with the PGE electrode connected to the negative terminal. After 6 minutes, the ITO electrode was allowed to dry and was inspected visually. The electrode was rotated and placed back in the solution with the same voltage applied for an additional 4 minutes. The electrode was inspected again, and deposition was carried out for another 2 minutes if required. The electrode was rinsed with water before use.

Enzyme loading

Enzymes were loaded into nanoporous ITO electrodes as reported.[1,4] In brief, a concentrated enzyme solution (~4 µL) was dropped onto the electrode surface and allowed to incubate for 30–45 minutes at room temperature. The electrode was then rinsed thoroughly with buffer to ensure that any non-adsorbed/trapped enzyme was removed. For the IDH1 R132H inhibition experiments, 0.85 nmoles of IDH1 R132H (dimer basis) was used; the amount of co-loaded FNR and/or wildtype IDH1 was adjusted to achieve the desired molar loading ratio.

Electrochemical experiments

Electrochemical experiments were performed as reported[4] using an Autolab PGSTAT 10 potentiostat and Nova software. All experiments were performed in an anaerobic glove box (Braun Technologies) containing a nitrogen atmosphere (O_2 < 1 ppm). A two-chamber glass electrochemical cell was used for all experiments: the ITO/PGE rotating disc electrode (working electrode) and platinum counter electrode were present in the same chamber, while the reference electrode was housed in a separate chamber (connected by a Luggin capillary) containing 0.1 M NaCl.[1,3,4] Electrode potentials were measured against a saturated calomel electrode (SCE) and converted to SHE using a conversion table.[1]

IDH1 R132H inhibition timecourse (chronoamperometry) experiments

IDH1 R132H and FNR were loaded (see "enzyme loading") onto a 0.06 cm^2 ITO/PGE rotating disc electrode using a molar loading ratio of FNR:IDH1 R132H; 1:2.5. A buffered solution (pH = 8) containing 20 mM each: MES, TAPS, CHES and 10 µM NADPH was used for all timecourse inhibition experiments. Concentrations of $MgCl_2$ and 2OG were varied. The electrode was rotated at a rate of 1000 rpm, the temperature was maintained at 25 °C, and an electrode potential of –0.5 V vs SHE was applied for the duration of the experiment. The bulk solution reaction volume was 4 mL. The IDH1 R132H reaction (2OG reduction to 2HG coupled to NADPH oxidation) was initiated by the addition of substrate (2-oxoglutarate). Once a steady-state reaction rate was achieved, inhibitor (in DMSO) was injected into the bulk solution (at t = 0) and the rate of decrease in enzyme activity (the rate of the inhibition reaction) was monitored. All inhibition experiments were carried out under anaerobic conditions (O_2 < 1 ppm).

Solution enzyme inhibition kinetic assays

Inhibition experiments were carried out in homogeneous solution for comparison with inhibition experiments measured under the nanoconfined conditions in the e-Leaf. The IDH1 R132H reaction rate (reduction of 2OG to 2HG coupled to NADPH oxidation) was monitored by measuring the change in NADPH concentration (absorbance at 340 nm) over time via UV-vis spectroscopy (PerkinElmer UV/VIS/NIR Lambda 19 Spectrometer). The reaction was carried out under nearly identical conditions to the electrochemical inhibition experiments: the solution had a pH = 8 (20 mM each: MES, TAPS, CHES) and contained 10 mM $MgCl_2$, 1 mM 2OG, and 100 µM NADPH. The reaction temperature was 25 °C and was initiated by addition

of 100 nM IDH1 R132H (concentration based on the dimeric form). A concentration of 100 nM IDH1 R132H was chosen because it afforded a long-lived steady-state reaction that should allow the effect of a slow-acting inhibitor to be observed (i.e., 100 nM enzyme gave a near constant reaction rate for > 30 minutes). Once a steady-state reaction rate was achieved (i.e. the decrease in [NADPH] was linear), the cuvette was removed, and inhibitor was added (1 µL solution in DMSO at $t = 0$); the solution was mixed, and measurement was resumed. Control experiments had DMSO added without inhibitor.

Treatment of primary data: assumptions required for obtaining inhibition kinetic parameters

In order to derive useful kinetic information on inhibition from a typical chronoamperometry experiment (measuring electric current over time at a fixed applied potential), two assumptions must be made to allow current to act as a proxy for the amount of active IDH1 R312H present in the electrode (as shown in **Figure 3A** in the main text): (1) the measured current is directly proportional to the rate of IDH1 R132H catalysis under conditions where IDH1 R132H activity is limiting; (2) the specific activity (turnover rate) of each IDH1 R132H molecule is roughly equal throughout the 3–6 µM thick nanoporous ITO layer under the steady-state conditions achieved using a rotating disc electrode. Assumption 1 has previously been shown to be correct for IDH1 R132H[1] and several other E2 enzymes.[4–6] The second assumption is supported by a recent modelling study showing that, for an E2 enzyme with a dilute solution $k_{cat} = 1000\ s^{-1}$, the activities of the most deeply buried and the least buried enzyme molecules should be the same within a 5 µm-thick nanoporous ITO electrode, i.e., an enzyme molecule buried at 5 µm depth has the same turnover rate as an enzyme molecule near the electrode surface;[7] the solution k_{cat} of IDH1 R132H is approximately 1–2 s^{-1},[2,8,9] thus the activity profile of R132H molecules can be assumed to be approximately homogeneous within the nanoporous electrode. With these two assumptions fulfilled, the measured current is thus proportional to the amount of active IDH1 R132H in the electrode nanopores.

The final crucial point enabling analysis is that, even at nanomolar concentrations, the inhibitor in the 4 mL bulk solution is present at much higher absolute quantities (0.4–80 nmoles for concentrations of 100 nM to 20 µM, respectively) than the concentrated IDH1 R132H enzyme that is trapped within the electrode nanopores (around 17 pmoles).[4] Because of this, the inhibitor is maintained at an effectively constant concentration throughout the experiment. With sufficient electrode rotation to maintain a constant local inhibitor concentration at the electrode, the reaction will exhibit pseudo first-order kinetics and can be analyzed accordingly.

Determining k_{obs} from timecourse experiments

Observed rate constant values (k_{obs}) for the inhibition of IDH1 R132H were determined by fitting a first-order exponential decay equation ("ExpDec1" fit using OriginLabs software) to chronoamperometry experiments that showed the rate of loss of enzyme activity over time (see "**treatment of primary data**" above for details on how the measured electrical current is directly proportional to the amount of active IDH1 R132H enzyme present). The exponential decay equation was fit to the data for at least two half-lives of inhibition. The primary data were "film-loss corrected" (see below for details) before curve fitting to correct for the slight loss in enzyme activity during the course of an experiment. Obtaining k_{obs} values via direct curve fitting was preferred over using the slope of the semi-log plot (example shown in **Figure 3B** in the main text) as it was both more convenient and more reliable than the slope method: curve fitting does not require the equilibrium concentration of free enzyme ($[E]_\infty$) to be known, and this value is convoluted by the film-loss observed during experiments. Determining the k_{obs} using curve fitting is similar to the Guggenheim method,[10–12] which does not require

equilibrium values to be known. Guggenheim analysis was also performed on the electrochemical inhibition data for comparison; rate constants measured using this alternative method were virtually identical to those obtained via direct curve fitting.

Film-loss correction

Prior to curve fitting to obtain k_{obs} values, the inhibition timecourse (chronoamperometry) data were corrected to account for the small background decrease in enzyme activity over time (termed “film loss” in protein film electrochemistry). This was done despite the background loss in activity only having a marginal effect on measured kinetic constants (similar values are obtained when fitting the corrected and uncorrected data). To do this, eight control experiments were conducted at different 2OG and Mg^{2+} concentrations with DMSO injected into the solution (no inhibitor) to measure the average background loss in activity over time. The background activity loss did not correlate with different concentrations of 2OG or Mg^{2+} and was roughly linear after steady-state enzyme activity was established. The average decrease in activity over a 5-hour period for eight control experiments was 44 ± 5% (i.e., 56% activity remained after 5 hours on average). To correct for this, the average decay rate per data point measured (every 3 seconds) was added back as a percentage of the current measured at that timepoint. In other words, every three seconds, the background decrease in activity was 0.007375%; this value was multiplied by the current measured at each timepoint, and this new value was added to the next current data point measured, in a cumulative fashion (i.e., each preceding background decay data point calculation was also added to the raw data point to obtain the corrected data point). Despite the background activity loss not having a large effect on measured kinetic parameters, the correction method proved effective, with control experiments maintaining a percent activity near 100% for > 2 hours—importantly, most inhibition kinetic data were obtained in less than one hour.

As noted above, the data correction did not substantially affect the measured rate constants. This is because the most important data was obtained within the first 20–60 minutes of measurement (depending on inhibitor/2OG/Mg^{2+} concentration) during which time control experiments lost an average of only 3–9% of activity. Importantly, because film loss appears as a roughly linear percentage of activity lost over time, and because the (exponential) rate of enzyme inhibition is fastest just after injection of inhibitor (when [E] is highest), the percentage of total initial enzyme activity lost due to film loss for inhibition experiments is far less than that for control experiments (i.e., film loss accounts for significantly less than 3–9% of the decrease in activity observed in inhibition experiments over the first 20–60 minutes).

The plot in **Figure 3A** (main text) was extended to 3.5 hours to show the approach to equilibrium, i.e., to show that inhibition is incomplete and there is active enzyme remaining. Although, the rate of film loss is low on the timescale of the initial enzyme inhibition, it does obscure visualization of the approach to equilibrium, hence we opted to correct the data. Importantly, measuring the observed rate constant (by fitting an exponential equation or Guggenheim analysis) does not require the equilibrium value to be known. **Figure S2** shows data before and after film-loss correction.

Supporting Results

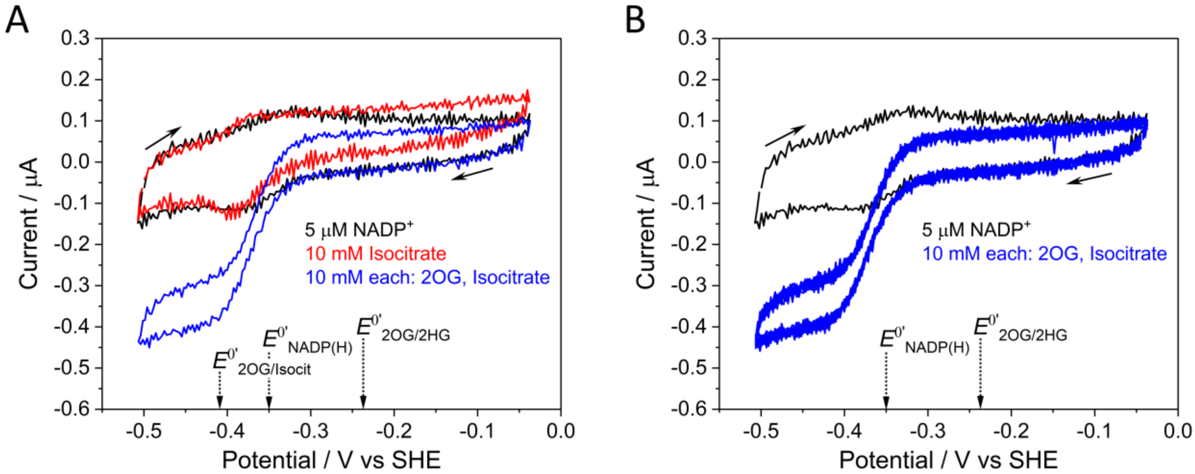

Figure S1. IDH1 R132H control experiments for the experiment presented in **Figure 2** in the main text. (A) The small amount of IDH1 R132H isocitrate oxidation activity is only detected when 2OG is not present. In this experiment, the isocitrate oxidation activity of IDH1 R132H was measured with 10 mM *DL*-isocitrate present; when 2OG is added, isocitrate oxidation activity is no longer detectable because IDH1 R132H immediately consumes the NADPH produced (from isocitrate oxidation) in the faster 2OG reduction reaction.[2] (B) Control experiment showing the stability of IDH1 R132H activity (2OG reduction) over time. The catalytic voltammograms (blue) shown in panel B are an overlay of 10 consecutive traces at a scan rate of 1 mV s^{-1} (including the first blue trace, which is shown in Panel A) equating to more than 2.6 hours of stable enzyme catalysis. Conditions: stationary (FNR+$IDH1_{R132H}$)@ITO/PGE electrode, electrode area 0.03 cm^2, scan rate 1 mV s^{-1}, temperature 25 °C, O_2 < 1 ppm, volume 4 mL, pH 8 (20 mM each: MES, TAPS, CHES), 10 mM $MgCl_2$, 5 μM $NADP^+$, enzyme loading ratios (molar): FNR/$IDH1_{R132H}$; 1/2.5.

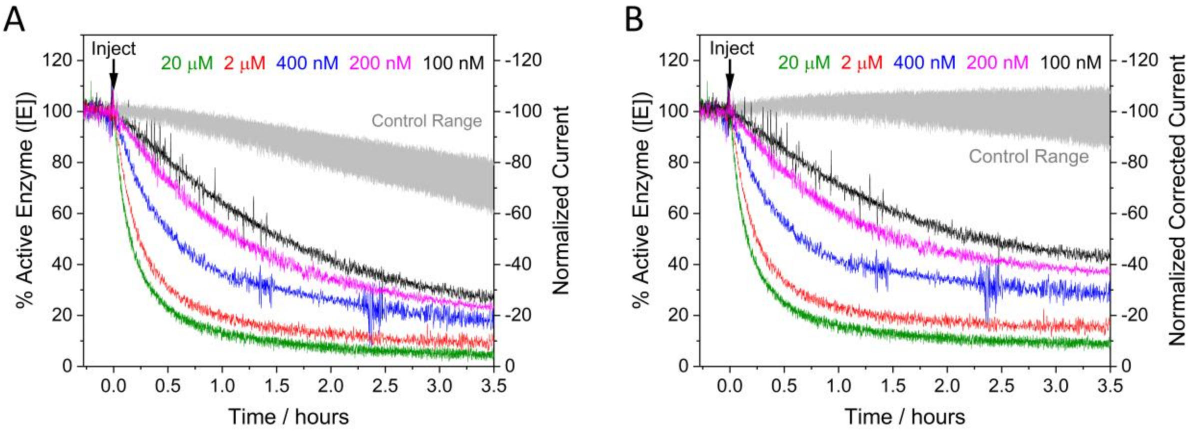

Figure S2. Plots of timecourse data before and after film loss correction. (A) Uncorrected inhibition timecourse data for the plot presented in **Figure 3A** in the main text (and panel B in this figure). (B) Plot of the data in panel A after correcting for film loss. Note that the main parameter affected by film loss correction is the equilibrium value; the data from which observed rate constants are most dependent (first 20–60 minutes) are largely unaffected by the data correction procedure (see **Materials and Methods** section on **Film-loss correction** for extended discussion).

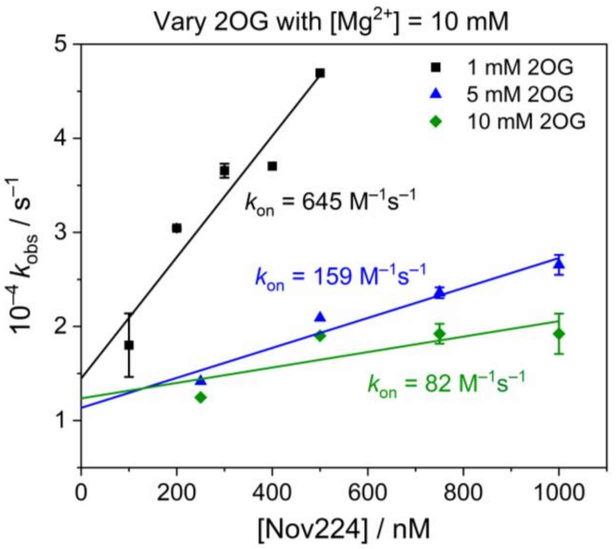

Figure S3. Pseudo first-order inhibition kinetics for Nov224 against IDH1 R132H at different concentrations of 2-oxoglutarate. The plotted k_{obs} values were determined by curve fitting a first-order exponential decay equation to timecourse inhibition experiments like those presented in **Figure 3A** in the main text. Conditions: (FNR+IDH1 R132H)@ITO/PGE electrode, electrode area 0.06 cm^2, rotation rate 1000 rpm, temperature 25 °C, E = –0.5 V vs SHE, O_2 < 1 ppm, volume 4 mL, pH = 8 (20 mM each of: MES, TAPS, CHES), 10 µM NADPH, 10 mM $MgCl_2$, enzyme loading ratios (molar): FNR/IDH1$_{R132H}$; 1/2.5.

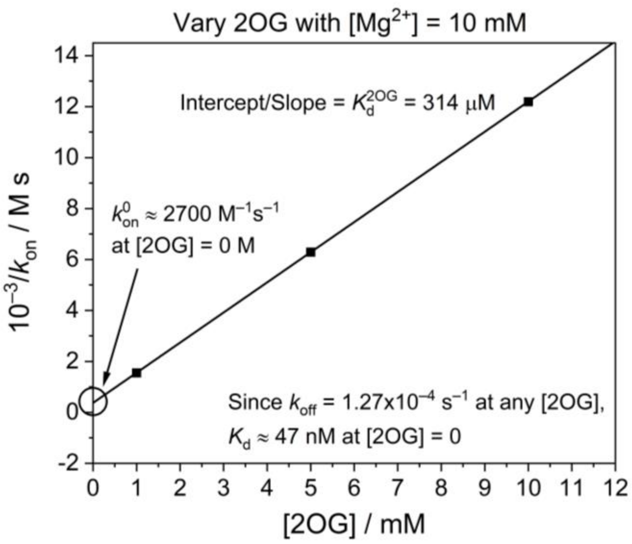

Figure S4. k_{on} data from the lines of best fit in **Figure S3** plotted using Eq. 6 (main text) to obtain limiting k_{on}^{0} values (and K_d values) for Nov224 where [2OG] and [Mg^{2+}] equal 0 M.

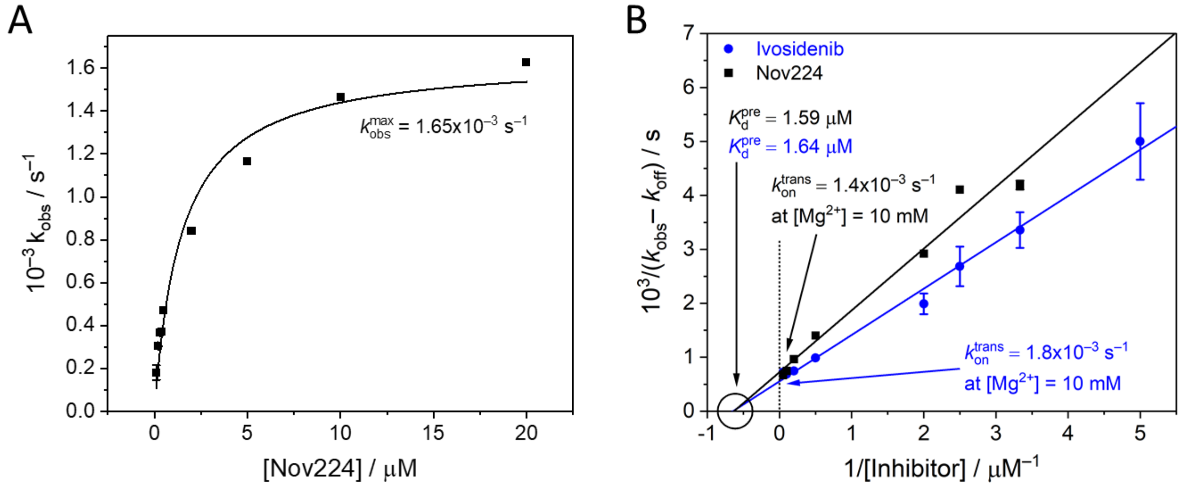

Figure S5. (A) Extended k_{obs} plot from the black data set presented in **Figure S3** ([2OG] = 1 mM) for different concentrations of Nov224 showing that the rate of inhibition reaches a limiting value at high inhibitor concentration. Conditions were otherwise the same as those for experiments with Ivosidenib. (B) Data from panel A plotted according to Eq. 11 (main text) to obtain k_{on}^{trans} and K_d^{pre} values for Nov224 (black) for comparison with data for Ivosidenib inhibiton (blue) at [2OG] = 1 mM, [Mg^{2+}] = 10 mM.

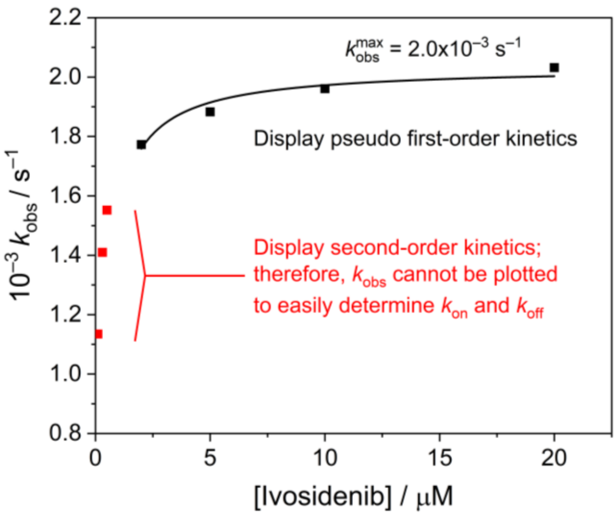

Figure S6. Plot of k_{obs} values (pH 8.0, temperature 25 ºC) against different Ivosidenib concentrations for inhibition experiments performed in dilute solution ([IDH1 R132H] = 100 nM). The k_{obs} values were determined by fitting a first-order exponential decay equation to enzyme rate vs time plots shown in **Figure 5B** in the main text. Note: experiments at 2 and 10 µM Ivosidenib were performed but are not shown in **Figure 5B** as they had roughly the same limiting rate of inhibition as 5 and 20 µM (as seen in this figure).

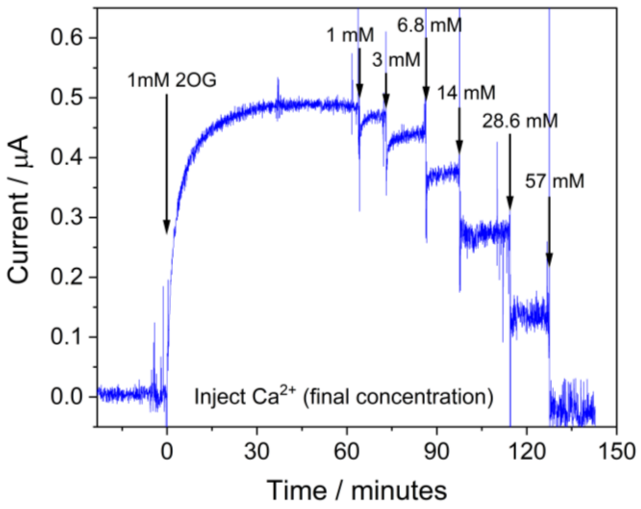

Figure S7. Control experiment showing fast inhibition of IDH1 R132H in the e-Leaf following injections of increasing concentrations of $CaCl_2$. This result demonstrates that the system is capable of responding to fast inhibition kinetics. Note that the concentrations shown represent the cumulative concentrations of $CaCl_2$ after each injection. Conditions: (FNR+IDH1 R132H)@ITO/PGE electrode, electrode area 0.06 cm^2, rotation rate 1000 rpm, temperature 25 °C, E = –0.5 V vs SHE, O_2 < 1 ppm, volume 4 mL, pH = 8 (20 mM each of: MES, TAPS, CHES), 10 µM NADPH, 10 mM $MgCl_2$, 1 mM 2OG, enzyme loading ratios (molar): FNR/$IDH1_{R132H}$; 1/2.5.

Supporting Analysis

Further testing the model

To test how well the data fit with the proposed two-step mechanism of inhibition, the overall inhibitor dissociation constant, K_d^I, was calculated by two different methods using data obtained at very high (extrapolated to infinity) Ivosidenib concentrations (**Figure 4D**) and data obtained at low (≤500 nM) Ivosidenib concentrations (**Figure 3D**). At low Ivosidenib concentrations, the K_d^I is simply the ratio k_{off}/k_{on}; in contrast, at high Ivosidenib concentration, the overall dissociation constant, K_d^I, is given by the product of the dissociation constants for each step:

$$K_d^I = \frac{k_{off} K_d^{pre}}{k_{on}^{trans}}. \qquad \text{(Eq. S1)}$$

When calculated using data at low Ivosidenib concentrations (**Figure 3D**), the K_d^I values are 76 nM and 156 nM at 3 mM and 10 mM Mg^{2+}, respectively; the corresponding K_d^I values obtained from experiments at high Ivosidenib concentrations (**Figure 4D**) are 65 nM and 136 nM at 3 mM and 10 mM Mg^{2+}, respectively. These values are in good agreement.

Supporting Table

Table S1. Comparing IDH inhibition kinetics (turnover conditions) and binding kinetics (non-turnover conditions).

Inhibitor	Enzyme	k_{on}/ $M^{-1} s^{-1}$	k_{off}/ s^{-1}	K_d/ µM	$[Mg^{2+}]$/ mM	[2OG]/ mM	Method	Turnover conditions	Ref.
**Ivosidenib**	**IDH1 R132H**	959	$1.50x10^{-4}$	0.156	10	1	e-Leaf	Yes	This work
**Nov224 (IDH224)**	**IDH1 R132H**	645	$1.27x10^{-4}$	0.197	10	1	e-Leaf	Yes	This work
**AG-221**	**IDH2 R140Q**	1480	$4.72x10^{-3}$	3.19	Not stated	Not stated	Solution	Yes	[13]
**Ivosidenib**	**IDH1 R132H**	322	$1.50x10^{-4}$	0.466	10	3.2	e-Leaf	Yes	This work
**Nov224 (IDH224)**	**IDH1 R132H**	241	$1.27x10^{-4}$	0.527	10	3.2	e-Leaf	Yes	This work
**AGI-6780**	**IDH2 R140Q**	967	$1.38x10^{-4}$	0.143	10	3.2	Solution	Yes	[14]
**Ivosidenib**	**IDH1 R132H**	8630	$1.50x10^{-4}$	0.017	10	0	e-Leaf	Yes	This work
**Nov224 (IDH224)**	**IDH1 R132H**	2700	$1.27x10^{-4}$	0.048	10	0	e-Leaf	Yes	This work
**ML309**	**IDH1 R132H**	32,000	$1.5x10^{-3}$	0.048	0	0	SPR	No	[15]

Note: Assays presented here using turnover conditions measure activity-based enzyme *inhibition* kinetics, whereas assays using non-turnover conditions (surface plasmon resonance (SPR)) measure the kinetics of binding/release; binding does not result in immediate inhibition in the case of IDH1 (see discussion and **Figure 6** in the main text). The $[Mg^{2+}]$ and [2OG] values are the concentrations under which inhibition/binding was measured. Ivosidenib values measured in this work were taken from **Figure 3C, D** and **Figure 4A, B** in the main text to match the 2OG/Mg^{2+} concentrations used in the cited studies. Nov224 values were obtained from **Figures S3–S4**.

Derivation of Equations

Derivation of the pseudo first-order reversible integrated rate law

For the reversible enzyme inhibition reaction

$$\mathrm{E} + \mathrm{I} \underset{k_{\mathrm{off}}}{\overset{k_{\mathrm{on}}}{\rightleftharpoons}} \mathrm{EI}\,, \qquad \text{(Eq. S2)}$$

the rate equation is

$$-\frac{d[\mathrm{E}]}{dt} = k_{\mathrm{on}}[\mathrm{E}][\mathrm{I}] - k_{\mathrm{off}}[\mathrm{EI}]\,. \qquad \text{(Eq. S3)}$$

Under pseudo first-order conditions where I is maintained at a constant concentration, the rate equation becomes

$$-\frac{d[\mathrm{E}]}{dt} = k_{\mathrm{on}}^{\mathrm{app}}[\mathrm{E}] - k_{\mathrm{off}}[\mathrm{EI}] \qquad \text{(Eq. S4)}$$

where

$$k_{\mathrm{on}}^{\mathrm{app}} = k_{\mathrm{on}}[\mathrm{I}]\,. \qquad \text{(Eq. S5)}$$

The pseudo first-order rate equation (Eq. 2) can then be treated in the same manner as a first-order reversible rate equation using the *apparent* first-order forward rate constant in place of the second-order forward rate constant. Therefore,

$$[\mathrm{E}]_{\mathrm{o}} = [\mathrm{E}]_t + [\mathrm{EI}]_t = [\mathrm{E}]_\infty + [\mathrm{EI}]_\infty\,, \qquad \text{(Eq. S6)}$$

and at equilibrium,

$$K = \frac{k_{\mathrm{on}}^{\mathrm{app}}}{k_{\mathrm{off}}} = \frac{[\mathrm{EI}]_\infty}{[\mathrm{E}]_\infty}\,, \qquad \text{(Eq. S7)}$$

where the subscripts o, t, and ∞ represent concentrations at time t = 0, t = variable, and t = infinity (concentration at equilibrium), respectively. Substituting Eq. S6 into Eq. S7 and solving for $[\mathrm{EI}]_t$ gives

$$[\mathrm{EI}]_t = \frac{k_{\mathrm{on}}^{\mathrm{app}}[\mathrm{E}]_\infty}{k_{\mathrm{off}}} + [\mathrm{E}]_\infty - [\mathrm{E}]_t\,. \qquad \text{(Eq. S8)}$$

Substituting Eq. S8 into Eq. S4 gives the rate law for a pseudo first-order reversible reaction:

$$-\frac{d[\mathrm{E}]}{dt} = ([\mathrm{E}]_t - [\mathrm{E}]_\infty)(k_{\mathrm{on}}^{\mathrm{app}} + k_{\mathrm{off}})\,. \qquad \text{(Eq. S9)}$$

Integration of Eq. S9 gives

$$\ln\left(\frac{[\mathrm{E}]_t - [\mathrm{E}]_\infty}{[\mathrm{E}]_{\mathrm{o}} - [\mathrm{E}]_\infty}\right) = -(k_{\mathrm{on}}^{\mathrm{app}} + k_{\mathrm{off}})t\,. \qquad \text{(Eq. S10)}$$

Substituting Eq. S5 into Eq. S10 gives the final integrated rate law for a pseudo first-order reversible reaction:

$$\ln\left(\frac{[\mathrm{E}]_t - [\mathrm{E}]_\infty}{[\mathrm{E}]_\mathrm{o} - [\mathrm{E}]_\infty}\right) = -(k_\mathrm{on}[\mathrm{I}] + k_\mathrm{off})t \quad \text{(Eq. S11)}$$

where

$$k_\mathrm{obs} = k_\mathrm{on}[\mathrm{I}] + k_\mathrm{off}\,. \quad \text{(Eq. S12)}$$

Therefore, plotting $\ln\left(\frac{[\mathrm{E}]_t - [\mathrm{E}]_\infty}{[\mathrm{E}]_\mathrm{o} - [\mathrm{E}]_\infty}\right)$ against t using gives a straight line with a slope of $-k_\mathrm{obs}$ (see **Figure 3B** in main text).

Derivation of rate law for competition between Ivosidenib and 2OG/Mg^{2+}

For the forward enzyme-inhibitor reaction (ignoring the slow dissociation step),

$$\mathrm{E} + \mathrm{I} \xrightarrow{k_\mathrm{on}} \mathrm{EI}\,, \quad \text{(Eq. S13)}$$

where the enzyme also binds rapidly and reversibly to agent X (X = 2OG or Mg^{2+}), preventing the inhibitor from reacting with the enzyme, according to

$$\mathrm{E} + \mathrm{X} \overset{K^\mathrm{X}}{\rightleftharpoons} \mathrm{EX}\,, \quad \text{(Eq. S14)}$$

then

$$K_\mathrm{d}^\mathrm{X} = \frac{[\mathrm{E}][\mathrm{X}]}{[\mathrm{EX}]}\,. \quad \text{(Eq. S15)}$$

Because

$$[\mathrm{EX}] = [\mathrm{E}]_\mathrm{o} - [\mathrm{E}]\,, \quad \text{(Eq. S16)}$$

therefore

$$K_\mathrm{d}^\mathrm{X} = \frac{[\mathrm{E}][\mathrm{X}]}{[\mathrm{E}]_\mathrm{o} - [\mathrm{E}]}\,. \quad \text{(Eq. S17)}$$

Rearranging gives

$$K_\mathrm{d}^\mathrm{X}[\mathrm{E}]_\mathrm{o} = [\mathrm{E}][\mathrm{X}] + K_\mathrm{d}^\mathrm{X}[\mathrm{E}] \quad \text{(Eq. S18)}$$

and solving for [E] gives

$$[\mathrm{E}] = \frac{K_\mathrm{d}^\mathrm{X}[\mathrm{E}]_\mathrm{o}}{K_\mathrm{d}^\mathrm{X} + [\mathrm{X}]}\,. \quad \text{(Eq. S19)}$$

Therefore, the rate of an enzyme-inhibitor reaction with a competing reaction between the enzyme and agent X is given by

$$\mathrm{rate} = k_\mathrm{on}[\mathrm{E}][\mathrm{I}] = \frac{k_\mathrm{on}K_\mathrm{d}^\mathrm{X}[\mathrm{E}]_\mathrm{o}[\mathrm{I}]}{K_\mathrm{d}^\mathrm{X} + [\mathrm{X}]}\,, \quad \text{(Eq. S20)}$$

where k_{on} is the observed second-order rate constant that depends on [X] which lowers the concentration of active enzyme. The form of the dependence (the attenuation of rate as [X] is increased) is obtained by noting that when [X] = 0, $k_{on} = k_{on}^{0}$. Therefore,

$$\text{rate} = k_{on}[\text{E}][\text{I}] = \frac{k_{on}^{0} K_{d}^{X} [\text{E}]_{o}[\text{I}]}{K_{d}^{X} + [\text{X}]} \quad , \qquad \text{(Eq. S21)}$$

and noting further that [E] = $[\text{E}]_o$ and dividing each side by $[\text{E}]_o[\text{I}]$ we obtain

$$k_{on} = \frac{k_{on}^{0} K_{d}^{X}}{K_{d}^{X} + [\text{X}]}, \qquad \text{(Eq. S22)}$$

in which k_{on} is independent of the point at which measurement is made, i.e., it is independent of [E]. The resulting rate equation thus has one dependent variable (k_{on}), one independent variable ([X]) and two constants (k_{on}^{0} and K_{d}^{X}). Setting [X] = 0 in Eq. S22, we see that $k_{on} = k_{on}^{0}$ as required.

Taking the reciprocal of Eq. S22 gives the linear equation:

$$\frac{1}{k_{on}} = \frac{1}{k_{on}^{0}} + \frac{[\text{X}]}{k_{on}^{0} K_{d}^{X}} . \qquad \text{(Eq. S23)}$$

Relating the observed rate constant to pre-equilibrium binding and the rate-determining inhibition step

Adopting the approach of Morrison and Walsh[16] for a two-step inhibition mechanism according to the reaction scheme

$$\text{E} + \text{I} \underset{k_2}{\overset{k_1}{\rightleftharpoons}} \text{EI} \underset{k_4}{\overset{k_3}{\rightleftharpoons}} \text{EI}^*, \qquad \text{(Eq. S24)}$$

where the first step is a rapid equilibrium (k_1, k_2 >> k_3, k_4) followed by a slow, intramolecular isomerization reaction, the observed rate constant is equal to

$$k_{obs} = \frac{k_3[\text{I}]}{K_i + [\text{I}]} + k_4 , \qquad \text{(Eq. S25)}$$

where $K_i = k_2/k_1$. Using the nomenclature presented in this work, and assuming that $k_{off}^{trans} = k_{off}$ (since k_{off}^{trans} will be rate-determining step for the dissociation process), Eq. S25 is equivalent to

$$k_{obs} = \frac{k_{on}^{trans}[\text{I}]}{K_{d}^{pre} + [\text{I}]} + k_{off} . \qquad \text{(Eq. S26)}$$

Rearranging gives

$$k_{obs} - k_{off} = \frac{k_{on}^{trans}[I]}{K_d^{pre} + [I]}, \qquad \text{(Eq. S27)}$$

and taking the reciprocal gives

$$\frac{1}{(k_{obs} - k_{off})} = \frac{1}{k_{on}^{trans}} + \frac{K_d^{pre}}{k_{on}^{trans}} \bullet \frac{1}{[I]}. \qquad \text{(Eq. S28)}$$

Therefore, a plot of $\frac{1}{(k_{obs} - k_{off})}$ versus 1/[I] gives a linear plot with a y-intercept = $1/k_{on}^{trans}$ and an x-intercept = $-1/K_d^{pre}$. An alternative method to derive Eq. S26 is given in the next section, where initially, the reverse kinetic step, k_{off}^{trans}, is ignored for simplicity (and because the reverse rate is very slow compared to the forward rate). This method yields Eq. S43, which is equivalent to Eq. S26 except that the y-intercept for Eq. S43 will equal 0 (because this equation does not account for the reverse reaction). To correct for this, the *off* rate constant, k_{off}, can be added to the right hand side of Eq. S43 so that the y-intercept is equal to the off rate constant (assuming that $k_{off}^{trans} = k_{off}$ since k_{off}^{trans} will be the rate-determining step in the reverse direction). Following this, Eq. S26 and Eq. S43 are equivalent, and Eq. S28 can be derived in the same manner as above.

Derivation of equations describing the hyperbolic dependence of the inhibition rate on [I]

The hyperbolic dependence of k_{obs} on [I], i.e., describing the rate reaching a limiting value, is given by the empirical formula

$$\text{rate}' = \frac{a[E][I]}{b + [I]}, \qquad \text{(Eq. S29)}$$

where rate´ ignores the small contribution from the off rate constant (the intercept obtained at low [I]). Since, for a pseudo first-order inhibition reaction (where [I] is constant),

$$\text{rate}' = k_{obs}[E], \qquad \text{(Eq. S30)}$$

we can obtain the pseudo first-order rate constant by dividing both sides of Eq. S30 by the enzyme concentration [E] (the value of k_{obs} does not depend on [E], but the term has to be extracted out) to give

$$k_{obs}' = \frac{a\,[I]}{b + [I]}, \qquad \text{(Eq. S31)}$$

where k_{obs}´ ignores the small contribution from the final off rate constant (the intercept obtained at low [I]). There are (at least) three ways that this rate law can arise:

(1) The inhibitor associates (rapidly) to form a non-inhibitory pre-equilibrium complex,

$$E + I \underset{}{\overset{K^{pre}}{\rightleftharpoons}} E \sim I, \qquad \text{(Eq. S32)}$$

which is followed by an intramolecular isomerization reaction that results in inhibition (this step is also reversible, although we simplify by ignoring the back reaction as the intercept correction is very small).

$$E \sim I \underset{k_{off}^{trans}}{\overset{k_{on}^{trans}}{\rightleftharpoons}} EI \qquad \text{(Eq. S33)}$$

Using the pre-equilibrium approach, the initial pre-equilibrium is given by

$$K_d^{pre} = \frac{[E][I]}{[E \sim I]} \qquad \text{(Eq. S34)}$$

Since

$$[E] = [E]_o - [E \sim I], \qquad \text{(Eq. S35)}$$

we obtain

$$K_d^{pre} = \frac{([E]_o - [E \sim I])[I]}{[E \sim I]} \qquad \text{(Eq. S36)}$$

and since

$$K_d^{pre}[E \sim I] = [E]_o[I] - [E \sim I][I] \qquad \text{(Eq. S37)}$$

and

$$K_d^{pre}[E \sim I] + [E \sim I][I] = [E]_o[I] \qquad \text{(Eq. S38)}$$

it follows that

$$[E \sim I] = \frac{[E]_o[I]}{K_d^{pre} + [I]}. \qquad \text{(Eq. S39)}$$

Since

$$\text{rate'} = k_{on}^{trans}[E \sim I], \qquad \text{(Eq. S40)}$$

The rate is given by

$$\text{rate'} = k_{on}^{trans}[E \sim I] = \frac{k_{on}^{trans}[E]_o[I]}{K_d^{pre} + [I]}. \qquad \text{(Eq. S41)}$$

The rate can also be described by:

$$\text{rate'} = k_{obs}[E] = \frac{k_{on}^{trans}[E]_o[I]}{K_d^{pre} + [I]}, \qquad \text{(Eq. S42)}$$

and since k_{obs} is independent of the point at which measurement is made, i.e., it is independent of [E], we obtain:

$$\text{rate}'/[\text{E}]_o = k_{obs} = \frac{k_{on}^{trans}[\text{I}]}{K_d^{pre} + [\text{I}]} \qquad \text{(Eq. S43)}$$

Thus, the terms a and b are as follows: $a = k_{on}^{trans}$ and $b = K_d^{pre}$.

(2) The inhibitor associates (more weakly) with the enzyme to form a complex that remains active and does not eventually become the inhibited state. The complex is thus a "dead end" complex,

$$\text{E} + \text{I} \overset{K_d^{\text{dead end}}}{\rightleftharpoons} \text{E} \sim \text{I} . \qquad \text{(Eq. S44)}$$

The free enzyme that remains reacts with I by another pathway to give the inhibited state in a second-order step

$$\text{E} + \text{I} \underset{k_{off}}{\overset{k_{on}}{\rightleftharpoons}} \text{EI} . \qquad \text{(Eq. S45)}$$

Using the competing equilibrium approach, the competing equilibrium is given by

$$K_d^{\text{dead end}} = \frac{[\text{E}][\text{I}]}{[\text{E} \sim \text{I}]} . \qquad \text{(Eq. S46)}$$

Since

$$[\text{E} \sim \text{I}] = [\text{E}]_o - [\text{E}] \qquad \text{(Eq. S47)}$$

We obtain

$$K_d^{\text{dead end}} = \frac{[\text{E}][\text{I}]}{[\text{E}]_o - [\text{E}]} \qquad \text{(Eq. S48)}$$

and since

$$K_d^{\text{dead end}}[\text{E}]_o - K_d^{\text{dead end}}[\text{E}] = [\text{E}][\text{I}] \qquad \text{(Eq. S49)}$$

and

$$K_d^{\text{dead end}}[\text{E}]_o = [\text{E}]([\text{I}] + K_d^{\text{dead end}}) \qquad \text{(Eq. S50)}$$

it follows that

$$[\text{E}] = \frac{K_d^{\text{dead end}}[\text{E}]_o}{K_d^{\text{dead end}} + [\text{I}]} . \qquad \text{(Eq. S51)}$$

Therefore,

$$\text{rate}' = k_{\text{on}}[\text{E}][\text{I}] = \frac{k_{\text{on}} K_{\text{d}}^{\text{dead end}} [\text{E}]_{\text{o}}[\text{I}]}{K_{\text{d}}^{\text{dead end}} + [\text{I}]} \qquad \text{(Eq. S52)}$$

As before, the rate can also be described by:

$$\text{rate}' = k_{\text{obs}}[\text{E}] = \frac{k_{\text{on}} K_{\text{d}}^{\text{dead end}} [\text{E}]_{\text{o}}[\text{I}]}{K_{\text{d}}^{\text{dead end}} + [\text{I}]}, \qquad \text{(Eq. S53)}$$

and since k_{obs} is independent of the point at which measurement is made, i.e., it is independent of [E], we obtain

$$\text{rate}'/[\text{E}] = k_{\text{obs}} = \frac{k_{\text{on}} K_{\text{d}}^{\text{dead end}} [\text{I}]}{K_{\text{d}}^{\text{dead end}} + [\text{I}]}. \qquad \text{(Eq. S54)}$$

Thus, the terms a and b are: $a = k_{\text{on}} K_{\text{d}}^{\text{dead end}}$ and $b = K_{\text{d}}^{\text{dead end}}$

(3) The initial state of the enzyme must first adopt an alternative conformation E* in a reaction given by forward and reverse rate constants $k_{\text{con+}}$ and $k_{\text{con-}}$

$$\text{E} \underset{k_{\text{con}-}}{\overset{k_{\text{con}+}}{\rightleftharpoons}} \text{E*} \qquad \text{(Eq. S55)}$$

where only the alternative conformation reacts with the inhibitor, in a second-order process

$$\text{E*} + \text{I} \underset{k_{\text{off}}}{\overset{k_{\text{on}}}{\rightleftharpoons}} \text{EI} \qquad \text{(Eq. S56)}$$

We use the steady-state approach. Note that since [E*] is now very small, it follows that [E] = $[\text{E}]_{\text{o}}$, i.e., there is no need to correct for the amount of complexed enzyme. Therefore,

$$\frac{d[\text{E*}]}{dt} = 0 = k_{\text{con+}}[\text{E}] - k_{\text{con-}}[\text{E*}] - k_{\text{on}}[\text{E*}][\text{I}]. \qquad \text{(Eq. S57)}$$

Thus,

$$[\text{E*}] = \frac{k_{\text{con+}}[\text{E}]}{k_{\text{con}-} + k_{\text{on}}[\text{I}]} \qquad \text{(Eq. S58)}$$

and

$$\text{rate}' = k_{\text{on}}[\text{E*}][\text{I}] = \frac{k_{\text{on}} k_{\text{con+}}[\text{E}][\text{I}]}{k_{\text{con}-} + k_{\text{on}}[\text{I}]}. \qquad \text{(Eq. S59)}$$

As before, the rate can also be described by:

$$\text{rate}' = k_{\text{obs}}[\text{E}] = \frac{k_{\text{on}} k_{\text{con+}} [\text{E}][\text{I}]}{k_{\text{con}-} + k_{\text{on}}[\text{I}]}, \qquad \text{(Eq. S60)}$$

and thus

$$\text{rate}'/[\text{E}] = k_{\text{obs}} = \frac{k_{\text{on}} k_{\text{con+}} [\text{I}]}{k_{\text{con}-} + k_{\text{on}}[\text{I}]}. \qquad \text{(Eq. S61)}$$

Finally, dividing each term in the right-hand side by k_{on} gives

$$\text{rate}'/[\text{E}] = k_{\text{obs}} = \frac{k_{\text{con+}} [\text{I}]}{\frac{k_{\text{con}-}}{k_{\text{on}}} + [\text{I}]}. \qquad \text{(Eq. S62)}$$

Thus, the terms a and b are as follows: $a = k_{\text{con+}}$, $b = k_{\text{con+}}/k_{\text{on}}$. In the context of IDH1 inhibition by Ivosidenib and other allosteric IDH inhibitors, option 3 can be ruled out as it does not account for any non-inhibitory binding modes (see main text for details).

References

[1] R. A. Herold, R. Reinbold, C. F. Megarity, M. I. Abboud, C. J. Schofield, F. A. Armstrong, *J. Phys. Chem. Lett.* **2021**, *12*, 6095–6101.

[2] R. Reinbold, I. C. Hvinden, P. Rabe, R. A. Herold, A. Finch, J. Wood, M. Morgan, M. Staudt, I. J. Clifton, F. A. Armstrong, J. S. O. McCullagh, J. Redmond, C. Bardella, M. I. Abboud, C. J. Schofield, *Nat. Commun.* **2022**, *13*, 4785.

[3] R. M. Evans, F. A. Armstrong, in *Methods Mol. Biol.*, Humana Press Inc., **2014**, pp. 73–94.

[4] R. A. Herold, R. Reinbold, C. J. Schofield, F. A. Armstrong, *Proc. Natl. Acad. Sci.* **2023**, *120*, e2214123120.

[5] C. F. Megarity, B. Siritanaratkul, R. S. Heath, L. Wan, G. Morello, S. R. FitzPatrick, R. L. Booth, A. J. Sills, A. W. Robertson, J. H. Warner, N. J. Turner, F. A. Armstrong, *Angew. Chemie Int. Ed.* **2019**, *58*, 4948–4952.

[6] F. A. Armstrong, B. Cheng, R. A. Herold, C. F. Megarity, B. Siritanaratkul, *Chem. Rev.* **2023**, *123*, 5421–5458.

[7] B. Siritanaratkul, *Phys. Chem. Chem. Phys.* **2023**, *25*, 9357–9363.

[8] X. Liu, R. Reinbold, S. Liu, R. A. Herold, P. Rabe, S. Duclos, R. B. Yadav, M. I. Abboud, S. Thieffine, F. A. Armstrong, L. Brewitz, C. J. Schofield, *J. Biol. Chem.* **2023**, *299*, 102873.

[9] S. Liu, M. I. Abboud, T. John, V. Mikhailov, I. Hvinden, J. Walsby-Tickle, X. Liu, I. Pettinati, T. Cadoux-Hudson, J. S. O. McCullagh, C. J. Schofield, *Commun. Biol. 2021 41* **2021**, *4*, 1–16.

[10] E. A. Guggenheim, *London, Edinburgh, Dublin Philos. Mag. J. Sci.* **1926**, *2*, 538–543.

[11] P. J. Niebergall, E. T. Sugita, *J. Pharm. Sci.* **1968**, *57*, 1805–1808.

[12] A. Cornish-Bowden, *Fundamentals of Enzyme Kinetics*, Wiley, **2012**.

[13] K. Yen, J. Travins, F. Wang, M. D. David, E. Artin, K. Straley, A. Padyana, S. Gross, B. DeLaBarre, E. Tobin, Y. Chen, R. Nagaraja, S. Choe, L. Jin, Z. Konteatis, G. Cianchetta, J. O. Saunders, F. G. Salituro, C. Quivoron, P. Opolon, O. Bawa, V. Saada, A. Paci, S. Broutin, O. A. Bernard, S. de Botton, B. S. Marteyn, M. Pilichowska, Y. Xu, C. Fang, F. Jiang, W. Wei, S. Jin, L. Silverman, W. Liu, H. Yang, L. Dang, M. Dorsch, V. Penard-Lacronique, S. A. Biller, S.-S. M. Su, *Cancer Discov.* **2017**, *7*, 478–493.

[14] F. Wang, J. Travins, B. DeLaBarre, V. Penard-Lacronique, S. Schalm, E. Hansen, K. Straley, A. Kernytsky, W. Liu, C. Gliser, H. Yang, S. Gross, E. Artin, V. Saada, E. Mylonas, C. Quivoron, J. Popovici-Muller, J. O. Saunders, F. G. Salituro, S. Yan, S. Murray, W. Wei, Y. Gao, L. Dang, M. Dorsch, S. Agresta, D. P. Schenkein, S. A. Biller, S. M. Su, S. De Botton, K. E. Yen, *Science (80-.).* **2013**, *340*, 622–626.

[15] M. I. Davis, S. Gross, M. Shen, K. S. Straley, R. Pragani, W. A. Lea, J. Popovici-Muller, B. DeLaBarre, E. Artin, N. Thorne, D. S. Auld, Z. Li, L. Dang, M. B. Boxer, A. Simeonov, *J. Biol. Chem.* **2014**, *289*, 13717–13725.

[16] J. F. Morrison, C. T. Walsh, in *Adv. Enzymol. Relat. Areas Mol. Biol.*, John Wiley & Sons, Ltd, **1988**, pp. 201–301.
